# Frataxin deficiency lowers lean mass and triggers the integrated stress response in skeletal muscle

**DOI:** 10.1172/jci.insight.155201

**Published:** 2022-05-09

**Authors:** César Vásquez-Trincado, Julia Dunn, Ji In Han, Briyanna Hymms, Jaclyn Tamaroff, Monika Patel, Sara Nguyen, Anna Dedio, Kristin Wade, Chinazo Enigwe, Zuzana Nichtova, David R. Lynch, Gyorgy Csordas, Shana E. McCormack, Erin L. Seifert

**Affiliations:** 1 Department of Pathology, Anatomy, and Cell Biology, Sidney Kimmel Medical College and; 2 MitoCare Center, Thomas Jefferson University, Philadelphia, Pennsylvania, USA.; 3 Division of Endocrinology and Diabetes and; 4 Division of Neurology, Children’s Hospital of Philadelphia, Philadelphia, Pennsylvania, USA.; 5 Department of Neurology and; 6 Department of Pediatrics, Perelman School of Medicine, University of Pennsylvania, Philadelphia, Pennsylvania, USA.

**Keywords:** Muscle Biology, Cell stress, Mitochondria, Skeletal muscle

## Abstract

Friedreich’s ataxia (FRDA) is an inherited disorder caused by reduced levels of frataxin (FXN), which is required for iron-sulfur cluster biogenesis. Neurological and cardiac comorbidities are prominent and have been a major focus of study. Skeletal muscle has received less attention despite indications that FXN loss affects it. Here, we show that lean mass is lower, whereas body mass index is unaltered, in separate cohorts of adults and children with FRDA. In adults, lower lean mass correlated with disease severity. To further investigate FXN loss in skeletal muscle, we used a transgenic mouse model of whole-body inducible and progressive FXN depletion. There was little impact of FXN loss when FXN was approximately 20% of control levels. When residual FXN was approximately 5% of control levels, muscle mass was lower along with absolute grip strength. When we examined mechanisms that can affect muscle mass, only global protein translation was lower, accompanied by integrated stress response (ISR) activation. Also in mice, aerobic exercise training, initiated prior to the muscle mass difference, improved running capacity, yet, muscle mass and the ISR remained as in untrained mice. Thus, FXN loss can lead to lower lean mass, with ISR activation, both of which are insensitive to exercise training.

## Graphical abstract

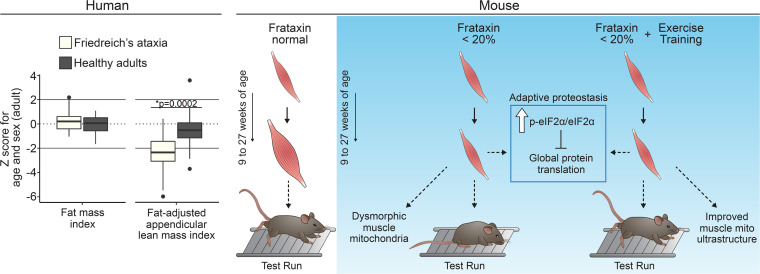

## Introduction

Friedreich’s ataxia (FRDA) is caused by the loss of frataxin protein (FXN), due, in most cases, to a homozygous GAA expansion within the first intron of the *Fxn* gene encoding FXN. The extent of FXN depletion depends on the length of the GAA expansion, which also generally dictates disease severity (1, 2). Neurological deficits define FRDA and strongly correlate with GAA repeat number (1). Cardiomyopathy also strongly correlates with GAA repeat number and is the leading cause of premature mortality (3).

Skeletal muscle has not been considered a major source of morbidity in FRDA. Yet, ATP-generating capacity has been shown to be lower in the skeletal muscle of individuals with FRDA (4–7), and of FXN-depleted mice (8), and strongly correlates with GAA repeat number (5, 6). Furthermore, muscle biopsies revealed that the activity of some electron transport chain complexes was at the lower end of the reference range; this was found most consistently for complex I (9, 10). Cytochrome *c*–negative fibers were also detected, as were fibers with increased subsarcolemmal succinate dehydrogenase activity (10) reminiscent of an expansion of the subsarcolemmal mitochondrial population that can be observed in primary mitochondrial myopathy (11). That oxidative phosphorylation (oxphos) capacity can be lower is not surprising given FXN is required for the biogenesis of iron-sulfur clusters (ISCs), and the function of several proteins needed for substrate catabolism and oxphos depends on ISCs (e.g., complex I contains 8 ISCs and cytochrome *c* contains heme, which depends on ISC-containing ferrochelatase for biosynthesis) (12). Overall, these observations indicate that the population of mitochondria in the skeletal muscle of individuals with FRDA can harbor functional defects.

A cohort of genetically and clinically diagnosed individuals with FRDA (*n* = 140) demonstrated a substantial prevalence of skeletal muscle weakness and wasting (1). The incidence of both conditions was correlated with GAA expansion length, albeit not as strongly cardiomyopathy and neurological phenotypes correlated to GAA repeat number (1). Whether muscle wasting was related to other disease features was not investigated, and muscle wasting was noted on clinical examination only, i.e., not quantified. Myofibers with smaller cross-sectional area (CSA) have been directly observed in muscle biopsies from a small cohort of patients with FRDA (10). These observations indicate that FXN-depleted skeletal muscle can be altered beyond changes in mitochondria. Furthermore, while lower oxphos capacity would be a plausible explanation for muscle weakness, the observations of smaller myofiber CSA (10) and wasting (1) raise the possibility that muscle weakness reflects decreased myofiber CSA. However, little attention has been given to potential muscle mass/growth changes in the context of FXN depletion. To investigate the effects of FXN deficiency on muscle, we first measured body composition in a genetically and clinically defined cohort of children and adults with FRDA and found lower lean mass in arms and legs, likely reflecting less skeletal muscle. To enrich our understanding, we next measured skeletal muscle mass using a recently described mouse model of induced progressive FXN depletion and found suppressed muscle mass gain. To gain mechanistic insights into the muscle-intrinsic factors that may have contributed, we analyzed proteostasis in FXN-depleted muscle and found that the integrated stress response (ISR) was activated, and global protein translation was lower. Finally, because physical activity provides a general anabolic stimulus, and in light of ongoing exercise trials in individuals with FRDA, mice were challenged with daily treadmill running to determine whether regular exercise could mitigate the muscle growth defect in FXN-depleted skeletal muscle. Though daily exercise improved running capacity on an inclined treadmill, it did not prevent muscle mass from being lower or the ISR activation.

## Results

### Lean mass is lower in adults with FRDA and correlates with disease severity.

**Figure 1:**
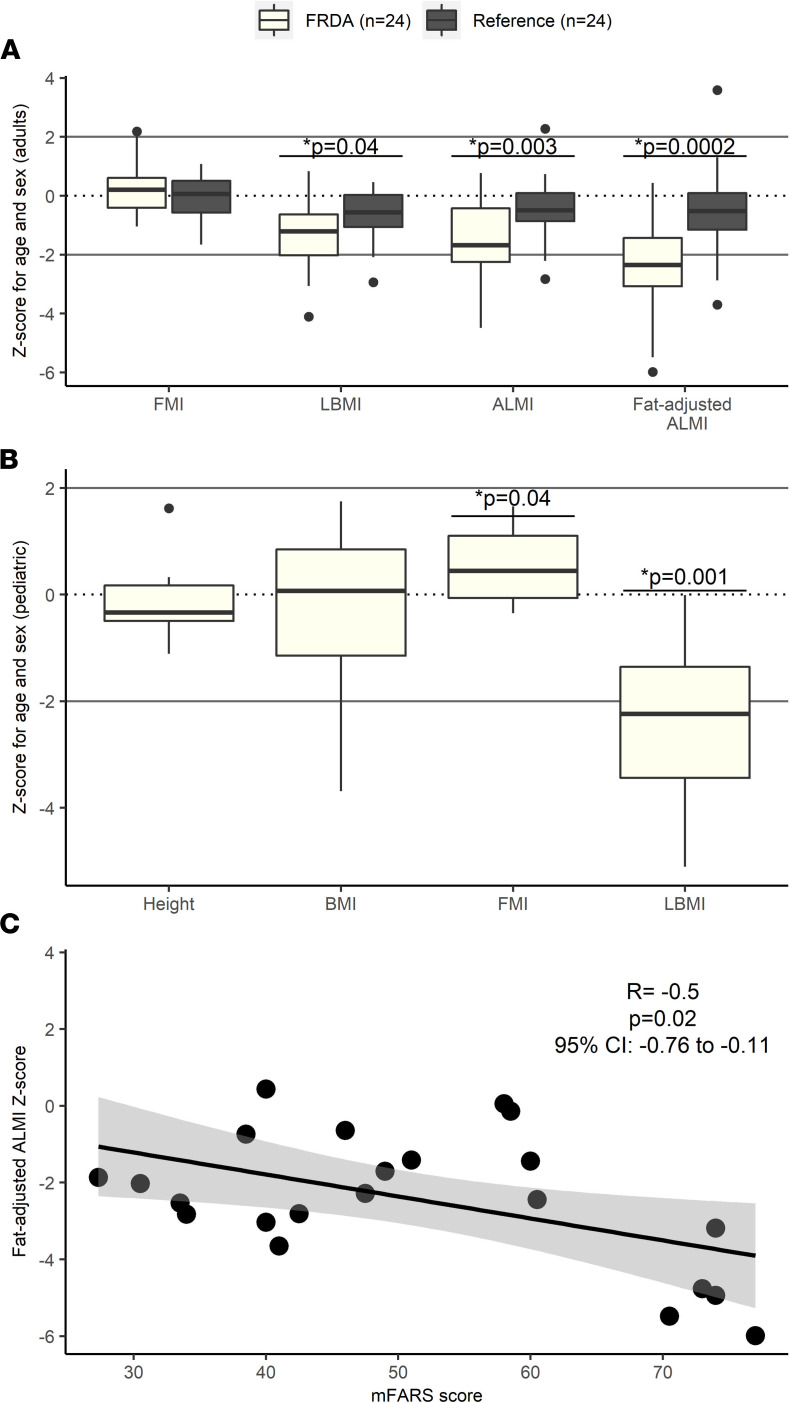
Lower lean mass in FRDA adults correlates with increased FRDA clinical disease severity. (**A**) Body composition *z* scores in healthy adults (*n* = 24) and adults with FRDA (*n* = 24). Two-tailed *t* tests between the 2 groups revealed that while there was no difference between the fat mass index (FMI) *z* scores in adults with and without FRDA, lean BMI (LBMI) was lower in adults with FRDA (–1.31 ± 1.21) compared with those without (–0.67 ± 0.87) (difference of –0.64; 95% CI, –1.25 to –0.02; *P* = 0.04). The same was true for appendicular lean mass index (ALMI) *z* scores, which were –1.61 ± 1.38 for individuals with FRDA and –0.5 ± 1.02 for those without (difference of –1.11; 95% CI, –1.82 to –0.41, *P* = 0.003), and for ALMI adjusted for fat mass, which were –2.41 ± 1.69 for adults with FRDA and –0.55 ± 1.47 for adults without FRDA (difference of –1.86; 95% CI, –2.78 to –0.95; *P* = 0.0002). (**B**) Body composition *z* scores in pediatric participants with FRDA (*n* = 10). One-tailed *t* tests comparing *z* scores from children with FRDA to 0, the expected mean *z* score based on the reference population, revealed no difference between the height and BMI *z* scores of children with FRDA and a population of healthy children with an average *z* score of 0. FMI was higher in children with FRDA (0.56 ± 0.73, 95% CI, 0.04 to 1.08; *P* = 0.04) while LBMI was lower than expected in children with FRDA (–2.45 ± 1.65, 95% CI, –3.63 to –1.27; *P* = 0.001). The box plots depict the minimum and maximum values (whiskers), the upper and lower quartiles, and the median. The length of the box represents the interquartile range. (**C**) Pearson’s correlation between fat-adjusted ALMI *z* score and modified Friedreich’s Ataxia Rating Scale (mFARS) scores, a marker of clinical severity, in adults with FRDA. *R* = –0.5; 95% CI, –0.76 to –0.11; *P* = 0.02.

Body composition variables were measured using dual x-ray absorptiometry (DXA) scans and compared between healthy adults and age-matched adults with FRDA (exclusion diagram in Supplemental Figure 1; supplemental material available online with this article; https://doi.org/10.1172/jci.insight.155201DS1) using standardized *z* scores. We found no difference between healthy adults and patients with FRDA in the median BMI (absolute values) or mean fat mass index *z* scores (Supplemental Table 1). In contrast, there were significant differences between the 2 groups in all 3 lean mass variables tested. Specifically, mean lean BMI, mean appendicular lean mass index, and fat-adjusted appendicular lean mass index *z* scores were lower in adults with FRDA (Figure 1A). Similar to adults, in children with FRDA, there was no significant difference in mean height or BMI *z* score compared to 0, which is the expected *z* score of a reference pediatric population. Conversely, the mean lean BMI *z* score was significantly lower in children with FRDA (Figure 1B) while the mean fat mass index *z* score was significantly higher. In adults, leg lean mass was nominally lower in individuals with FRDA versus without (Supplemental Figure 1B) (*P* = 0.05) while arm lean mass was similar (*P* = 0.47). For adults with FRDA, having a lower fat-adjusted appendicular lean mass index *z* score (i.e., less muscle in arms and legs) was correlated with a higher modified Friedreich’s Ataxia Rating Scale (mFARS) score (i.e., with greater disease severity) (Figure 1C). No correlation was detected between BMI and mFARS score. Additionally, no statistically significant correlations between body composition variables and other FRDA characteristics including GAA repeat length or disease duration were detected (not shown). In children, no statistically significant correlations between lean body mass variables and characteristics of FRDA were detected in this small pilot sample.

### Model of inducible FXN depletion.

**Figure 2:**
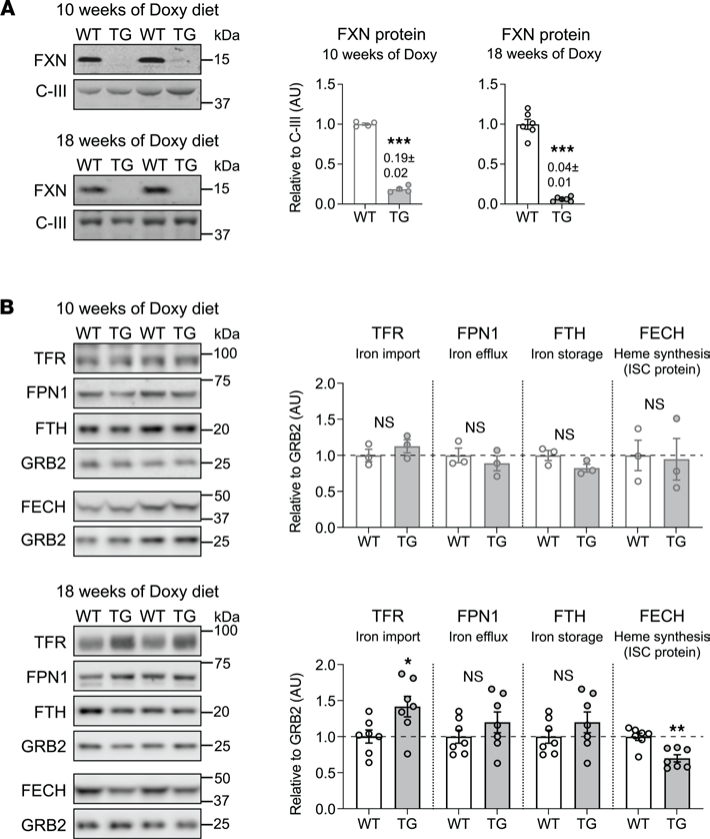
Mouse model of inducible FXN depletion leading to progressive loss of FXN in skeletal muscle. (**A**) *Left*: Representative immunoblots of FXN and GRB2 (loading control) in quadriceps lysates from WT and TG mice fed with Doxy diet for 10 and 18 weeks to induce whole-body FXN depletion. *Right*: Averaged values of FXN relative to GRB2 (*n* = 4/genotype for 10 weeks of Doxy diet; *n* = 6/genotype for 18 weeks of Doxy diet). Numbers in bar charts: mean ± SEM. (**B**) Evaluation of iron and heme handling/biosynthetic proteins by immunoblot in quadriceps lysates from WT and TG mice fed with Doxy for 10 weeks (*top*) and 18 weeks (*bottom*). Averaged values of each protein relative to GRB2 (loading control) are shown to the right of the corresponding immunoblots at 10 weeks of Doxy (*n* = 3/genotype) and 18 weeks of Doxy (*n* = 7/genotype). All panels: individual data points are shown, and bars represent mean ± SEM. Statistical comparison: unpaired *t* test, *P < 0.05, **P < 0.01, ***P < 0.001. TFR, transferrin receptor; FPN1, ferroportin1; FTH, ferritin; FECH, ferrochelatase.

To investigate how FXN loss can affect muscle mass, we took advantage of a recently developed mouse model of inducible whole-body Fxn depletion (13). We modified the model such that phenotypes could be observed over a longer time before substantial mortality was evident (14). FXN was depleted by feeding mice chow containing doxycycline (Doxy; 200 parts per million, ppm), starting at 9 weeks of age. Both transgenic (TG) mice (with a Doxy-inducible shRNA-containing transgene targeting *Fxn* mRNA) and WT littermates were fed the Doxy diet. Based on our previous findings (14), we focused our study on 2 time points: 10 and 18 weeks of Doxy feeding. TG mice fed with Doxy for 10 weeks had approximately 19% of the WT FXN protein amount in skeletal muscle (Figure 2A), and at 18 weeks of Doxy feeding, FXN protein in TG muscle was approximately 4% of the WT level (Figure 2A). For protein detection by immunoblot, we used the protein GRB2 as a loading control; GRB2 was validated as a loading control by comparing GRB2 expression to that of a nonspecific protein marker, MemCode (Supplemental Figure 2A; please see online supplement for all uncut blots). FXN deficiency is expected to directly affect the expression of ISC-containing proteins, is often associated with an iron homeostatic response, and can lead to iron accumulation, e.g., in the heart and brain (15). Thus, in skeletal muscle depleted of FXN, we first investigated these most basic parameters that can be associated with FXN loss. Skeletal muscle from mice fed with Doxy for 10 weeks showed no changes in levels of the major iron handling proteins, namely transferrin receptor (TFR), ferroportin1 (FPN1), and ferritin (FTH) or in the ISC-containing mitochondrial protein ferrochelatase (FECH) that is required for heme biosynthesis (Figure 2B). In contrast, at 18 weeks of Doxy feeding, skeletal muscle from TG mice exhibited greater protein levels of TFR (with no change in FPN1 and FTH) and a 25% decrease in FECH. TFR elevation is usually associated with a homeostatic response to low cellular iron levels (reviewed in ref. 12). In this context, Perls staining was used to check for iron accumulation (as ferric deposits); no such accumulation was evident in muscle from 18-week Doxy-fed TG mice (Supplemental Figure 2B, which also provides a positive control for the Perls stain). Similarly, lack of iron accumulation was documented in human muscle biopsies (9) and in quadriceps from the MCK mouse model of FRDA (16).

### Muscle growth impairment and proportionately lower strength in FXN-depleted mice.

**Figure 3:**
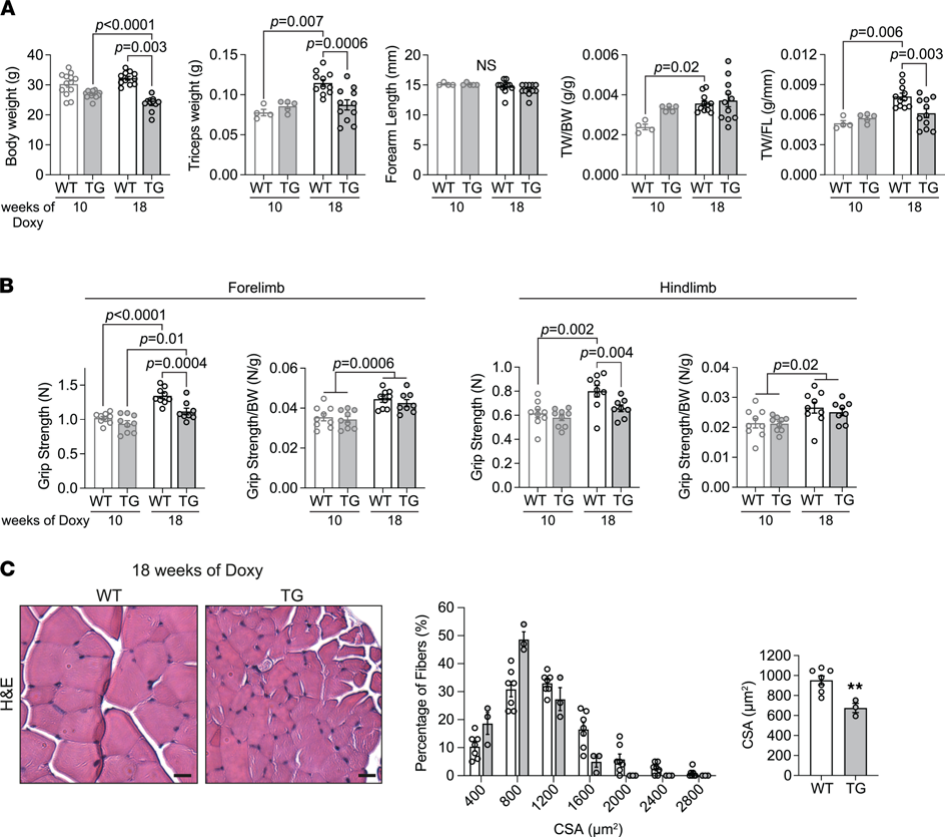
FXN depletion for 18 weeks induces an impairment in muscle growth and a proportionately lower grip strength. (**A**) Body weight (BW), triceps weight (TW), forearm length (FL), triceps weight to body weight ratio (TW/BW), and triceps weight to forearm length ratio (TW/FL) in WT and TG mice fed with Doxy diet for 10 weeks (*n* = 4 WT/5 TG for all the bar charts, except in BW, *n* = 13 WT/14 TG) and 18 weeks (*n* = 11 WT/11 TG for all bar charts). (**B**) Grip strength of the forelimbs and hind limbs, in absolute value and relative to the BW in mice fed Doxy for 10 weeks (*n* = 9 WT/9 TG) and 18 weeks (*n* = 9 WT/8 TG). (**C**) *Left*: Cross sections of EDL muscles from WT and TG mice fed with Doxy diet for 18 weeks, stained with H&E to evaluate cross-sectional area (CSA) of myofibers. Scale bars: 20 μm. *Center*: Distribution of myofiber CSA (125 fibers per muscle, *n* = 7 WT and 3 TG). *Right*: Averaged values of CSA. All panels: individual data points are shown, and bars represent mean ± SEM. (**A** and **B**) Statistics: 2-way ANOVA and post hoc comparisons using a Bonferroni correction (*P* values). (**C**) Statistical comparison: unpaired *t* test, **P < 0.01.

Next, we focused on skeletal muscle mass and strength. Comparing the 2 time points, we observed an increase in triceps weight (TW) in WT mice at 18 weeks compared with 10 weeks of Doxy feeding (Figure 3A). In contrast, there was no such change in FXN-depleted mice (Figure 3A), establishing a significant difference of the TW of WT and TG mice at 18 weeks. Changes in muscle mass were accompanied by a progressive decrease in BW in the TG mice (Figure 3A) (see also ref. 14). When TW was expressed as a ratio to BW (TW/BW), the difference between WT and TG at 18 weeks of Doxy feeding was abrogated (Figure 3A), indicating a proportional decrease in BW and TW. When TW was expressed as a ratio to the FL, the difference between WT and TG at 18 weeks of Doxy was evident (Figure 3A). Thus, TG mice specifically failed to increase muscle mass with age to the same extent as WT mice. To determine if TG mice inherently had smaller muscles than WT littermates, we measured TW/BW and TW/FL in mice that were not fed with Doxy and found no differences between WT and TG (Supplemental Figure 3A).

Muscle strength was evaluated in vivo as forelimb and hind limb grip strength. Grip strength did not differ between WT and TG mice fed Doxy for 10 weeks (Figure 3B). In contrast, the lower muscle mass in 18-week Doxy-fed TG mice was associated with lower forelimb and hind limb grip strength (Figure 3B). When grip strength was normalized to BW, both forelimb and hind limb grip strength were similar between WT and TG, and both genotypes showed a slight increase in normalized grip strength with age (Figure 3B). The latter results suggest that the lower muscle mass in TG mice fully accounted for their lower grip strength. The lower muscle mass in the FXN-depleted mice prompted us to analyze myofiber CSA and CSA distribution, which was done in extensor digitorum longus (EDL) muscle. Myofiber CSA was left shifted in TG EDL, and the average myofiber CSA was significantly smaller (Figure 3C), suggesting that the lower mass of muscle depleted of FXN was due to smaller myofiber size. Finally, we evaluated if there was a change in muscle fiber composition in the FXN-depleted mice. Immunoblot detection, using quadriceps lysates, of myosin heavy chain isoforms revealed no variation in fiber composition in TG versus control muscle (Supplemental Figure 3B).

### Lower global protein translation in FXN-depleted muscle.

**Figure 4:**
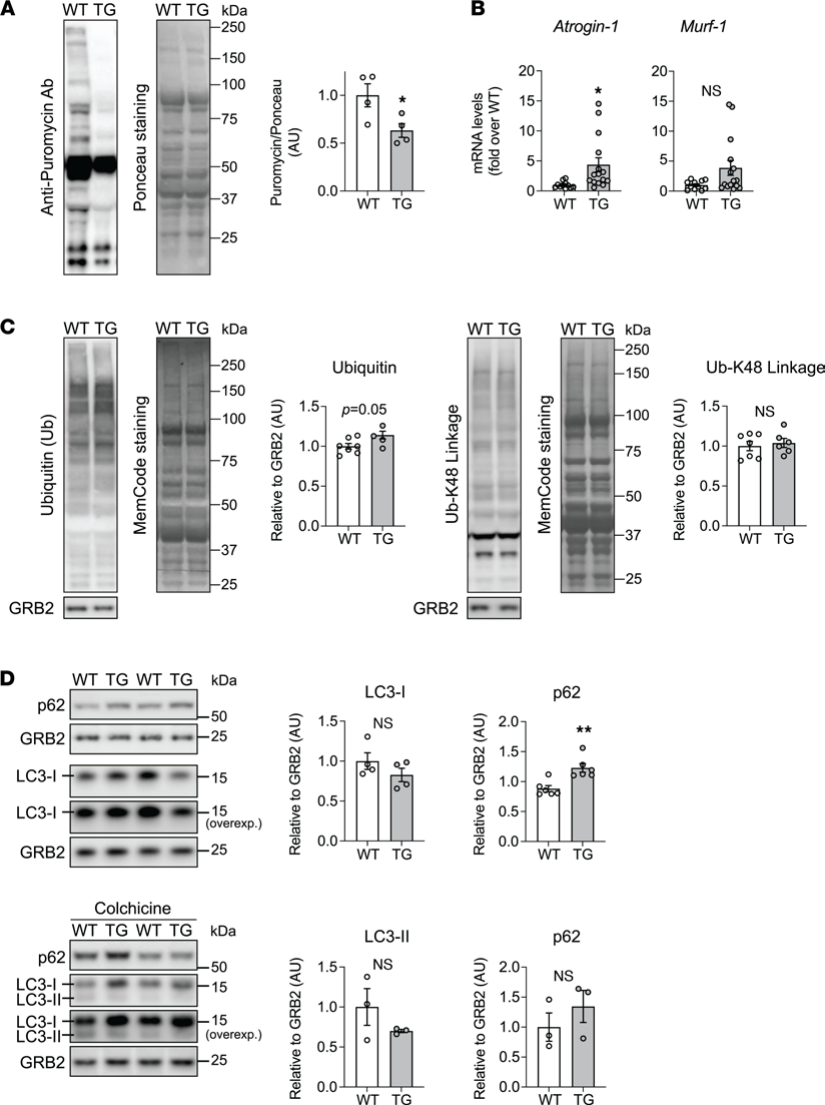
Muscle global protein translation is decreased after 18 weeks of FXN depletion. (**A**) Global protein translation was determined by the SUnSET method. *Left*: Representative immunoblot of puromycin-labeled proteins from tibialis anterior lysates of WT and TG mice fed with Doxy diet for 18 weeks. Ponceau Red staining was used to estimate total protein loading. *Right*: Quantification of the puromycin signal (*n* = 4/genotype). (**B**) Transcript levels of *Atrogin-1* (*Fbxo32*) and *MuRF-1* (*Trim63*) in quadriceps were normalized to *Actb* (β-actin) and expressed relative to WT (*n* = 11 WT/15 TG). (**C**) Representative immunoblots of total ubiquitin (Ub) and K-48–linked ubiquitination (Ub-K48) in quadriceps lysates from 18-week Doxy-fed mice. GRB2: loading control. Nonspecific membrane staining (MemCode) estimated protein loading. Averaged values of Ub/GRB2 and Ub-K48/GRB2 are shown on the right of each respective immunoblot (*n* = 7 WT/6 TG). (**D**) *Top*: Representative immunoblots of LC3 and p62 (GRB2: loading control). Quantification of LC3-I/GRB2 (*n* = 4 WT/5 TG) and p62/GRB2 (*n* = 6/genotype). *Bottom*: Representative immunoblots of LC3 and p62, from WT and TG mice treated with colchicine (0.4 mg/kg). Quantification of LC3-II/GRB2 and p62/GRB2 (*n* = 3/genotype). Quadriceps lysates were used. All panels: individual data points are shown, and bars represent mean ± SEM. Statistical comparison: unpaired *t* test, *P < 0.05, **P < 0.01.

Skeletal muscle mass is determined by a regulated balance between protein synthesis and breakdown (proteostasis) (17). Thus, we evaluated whether there was any dysregulation in muscle proteostasis under FXN depletion. First, in WT and TG mice fed with Doxy diet for 18 weeks, we measured global protein translation in vivo, using the SUnSET method of puromycin incorporation into elongating peptides and detection in muscle lysate by immunoblotting using an anti-puromycin antibody (18). Protein translation was significantly less in TG muscle (Figure 4A). To evaluate the status of protein degradation pathways, we first measured the mRNA levels of the 2 main E3 ubiquitin ligases, Atrogin-1 (*Fbxo32*) and MuRF-1 (*Trim63*). Atrogin1 mRNA was greater in muscle from TG 18-week Doxy-fed mice (Figure 4B), whereas MuRF1 transcript levels were unchanged (Figure 4B). After 10 weeks of Doxy diet, there were no differences in Atrogin1 and MuRF1 between WT and TG mice (Supplemental Figure 4A). We next determined the ubiquitination status of proteins by immunoblot. Total ubiquitin levels were slightly greater in muscle from 18-week Doxy-fed TG mice (Figure 4C), though the abundance of K-48– and K-63–linked polyubiquitin chains, which mark proteins for degradation and signal transduction, respectively (19, 20), was similar in WT and TG muscle (Figure 4C and Supplemental Figure 4B). Finally, to evaluate the status of macroautophagy, we measured protein levels of 2 autophagic markers, LC3 and p62, in quadriceps lysates. The level of p62 was higher in TG muscle; however, we were not able to detect the lipidated form of LC3, LC3-II (Figure 4D and Supplemental Figure 4C). To attempt to detect LC3-II, we used the microtubule depolarizing agent colchicine to stall autophagy by blocking autophagosome-to-autolysosome maturation (21). Using colchicine in vivo, LC3-II was detectable in quadriceps, but there was no significant difference in LC3-II, or in p62, between WT and TG (Figure 4D). Together, these results indicate that FXN loss disrupts proteostasis in skeletal muscle by decreasing protein synthesis.

### Activation of the ISR and mTORC1 signaling with FXN loss.

**Figure 5:**
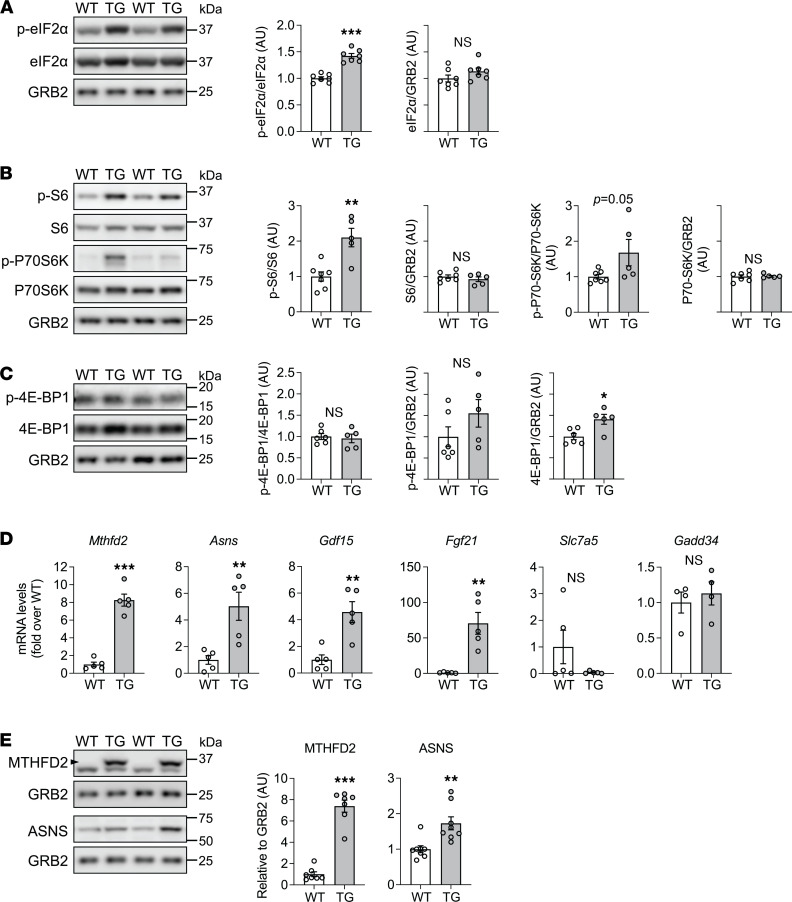
Activation of the ISR and elevated mTORC1 signaling in skeletal muscle after 18 weeks of FXN depletion. (**A**) *Left*: Representative immunoblots of p-eIF2α (Ser51), total eIF2α, and GRB2 (loading control). *Right*: Averaged values of p-eIF2α/eIF2α and eIF2α/GRB2 (*n* = 7/genotype). (**B**) *Left*: Representative immunoblots of p-S6 (Ser235/236), total S6, p–P70-S6K (Thr389), total P70-S6K, and GRB2 (loading control). *Right*: Quantification of p-S6/S6, S6/GRB2, p–P70-S6K/P70-S6K, and P70-S6K/GRB2 (*n* = 5 WT/7 TG). (**C**) *Left*: Representative immunoblots of p–4E-BP1 (Thr70), total 4E-BP1, and GRB2 (loading control). *Right*: Quantification of p–4E-BP1/4E-BP1, p–4E-BP1/GRB2, and 4E-BP1/GRB2 (*n* = 6 WT/5 TG). (**D**) Transcript levels (normalized to *Actb* and expressed relative to WT) of *Mthfd2*, *Asns*, *Gdf15*, *Fgf21*, *Slc7a5*, and *GADD34* (*n* = 4–5/genotype). (**E**) *Left*: Representative immunoblots of MTHFD2 (black arrowhead) and ASNS (GRB2: loading control). *Right*: Averaged values of MTHFD2/GRB2 and ASNS/GRB2 (*n* = 6–7/genotype). In all experiments, quadriceps lysates were used. In all panels: individual data points are shown, and bars represent mean ± SEM. Statistical comparison: unpaired *t* test, *P < 0.05, **P < 0.01, ***P < 0.001.

The lower protein translation in the skeletal muscle of 18-week Doxy-fed mice prompted us to evaluate 2 pathways implicated in the control of protein synthesis, namely the ISR and the mTORC1 pathway. The core event of ISR activation is the phosphorylation of the eukaryotic translation initiation factor eIF2α, leading to a suppression of global protein translation (22). On the other hand, mTORC1 signaling would stimulate protein synthesis (23). In TG muscles from mice fed with Doxy diet for 18 weeks, we found a marked increase in the level of phosphorylated (p-) eIF2α (Figure 5A). Interestingly, mTORC1 downstream targets, namely P70-S6K (ribosomal protein S6 kinase, 70 kDa) and S6 (ribosomal protein S6), were more phosphorylated in the FXN-depleted muscles (Figure 5B), suggesting mTORC1 pathway activation. Levels of p–4E-BP1, another downstream target of mTORC1, were not elevated in TG muscles when expressed relative to total 4E-BP1 or as a ratio to a loading control (GRB2), though total 4E-BP1 protein was elevated (Figure 5C). We also evaluated p-ULK1 (Ser757), an mTORC1-dependent phosphorylation target, but found no difference between WT and TG mice (Supplemental Figure 5A).

Activation of the ISR, and mTORC1 pathway, is related to increased protein levels of activating transcription factor 4 (ATF4) (22, 24, 25). We were, however, unable to detect ATF4, even in nucleus-enriched fractions (not shown), possibly due to a short half-life of the protein (~30–60 minutes; ref. 26). However, translation of ATF4 is expected to be increased when eIF2α is phosphorylated (25). Though we could not detect ATF4 protein, we could detect robust increases in transcripts of several ATF4 targets (Figure 5D), namely *Mthfd2* (1-carbon metabolism), *Asns* (amino acid metabolism), and *Gdf15* and *Fgf21* (both are secreted factors that alter metabolism through effects in the central nervous system and the liver, respectively), which have been found to be increased in primary mitochondrial myopathies (27–29). The ATF4 target *Slc7a5* (amino acid transporter), which we showed was elevated in the FXN-depleted heart from the mouse model used in the present study (14), was not increased in FXN-depleted skeletal muscle (Figure 5D). *GADD34* (*Ppp1r15a*) is also an ATF4 target and would provide negative feedback on the ISR (30); however, *GADD34* mRNA was not increased in TG muscle (Figure 5D). Additionally, protein levels of MTHFD2 and ASNS were substantially higher in the TG muscles (quadriceps) from 18-week Doxy-fed mice (Figure 5E). The higher MTHFD2 is noteworthy because this protein is normally not readily detected in skeletal muscle (Figure 5E). Moreover, 4E-BP1 is also an ATF4 target (31) and is elevated in FXN-depleted muscle (Figure 5C). Finally, we evaluated the activation of ISR in soleus, a slow twitch muscle with higher mitochondrial content relative to quadriceps (fast twitch). In comparison to quadriceps, p-eIF2α levels were similar in FXN-depleted soleus, with no difference in total eIF2α levels (Supplemental Figure 5). We also detected higher MTHFD2 levels in TG soleus, as we observed in TG quadriceps (Supplemental Figure 5).

Regarding the molecular drivers of the ISR and the elevated mTORC1 signaling in TG muscle from 18-week Doxy-fed mice, we confined our investigation to the activation status of major upstream kinases. Activated AKT and AMPK can be important sources of positive and negative regulation of mTORC1, respectively. Levels of p-AKT and p-AMPK, as well as AMPK targets (ACC and ULK1), were unchanged (Supplemental Figure 6, A and B). Regarding phosphorylation of eIF2α, 4 kinases have been identified, namely PERK, GCN2, HRI, and PKR (22), and activation status of 3 of these can be estimated using phospho-specific antibodies. We generated positive controls for each antibody (see Supplemental Figure 6C). We could not detect a difference between WT and TG muscle (from 18-week Doxy-fed mice) in the level of p-PERK (nor did the PERK signal show a gel shift indicative of increased phosphorylation) (Supplemental Figure 6C). However, we could detect elevated levels of binding immunoglobulin protein/GRP78 protein, indicating that ER stress was present (Supplemental Figure 6D). Phosphorylated GCN2 was only minimally detectable in skeletal muscle, and the level was similar between WT and TG muscles (Supplemental Figure 6C). HRI activation was evaluated under special electrophoretic conditions using a total HRI antibody (32) since a phospho-specific antibody is not available. We detected a band near the predicted molecular weight (MW) of 75 kDa; however, there was no evidence of a gel shift upward that would suggest phosphorylation, nor was band intensity greater (in fact, band intensity was lower, Supplemental Figure 6C). For p-PKR, we detected a band near 74 kDa in the skeletal muscle that differed from the MW of the signal of the positive control (68 kDa), which is also the predicted MW (Supplemental Figure 6C). Thus, among the canonical kinases that phosphorylate eIF2α, our findings suggest that we can rule out PERK and GCN2.

### FXN-depleted muscle exhibits alterations in mitochondrial function and ultrastructure.

**Figure 6:**
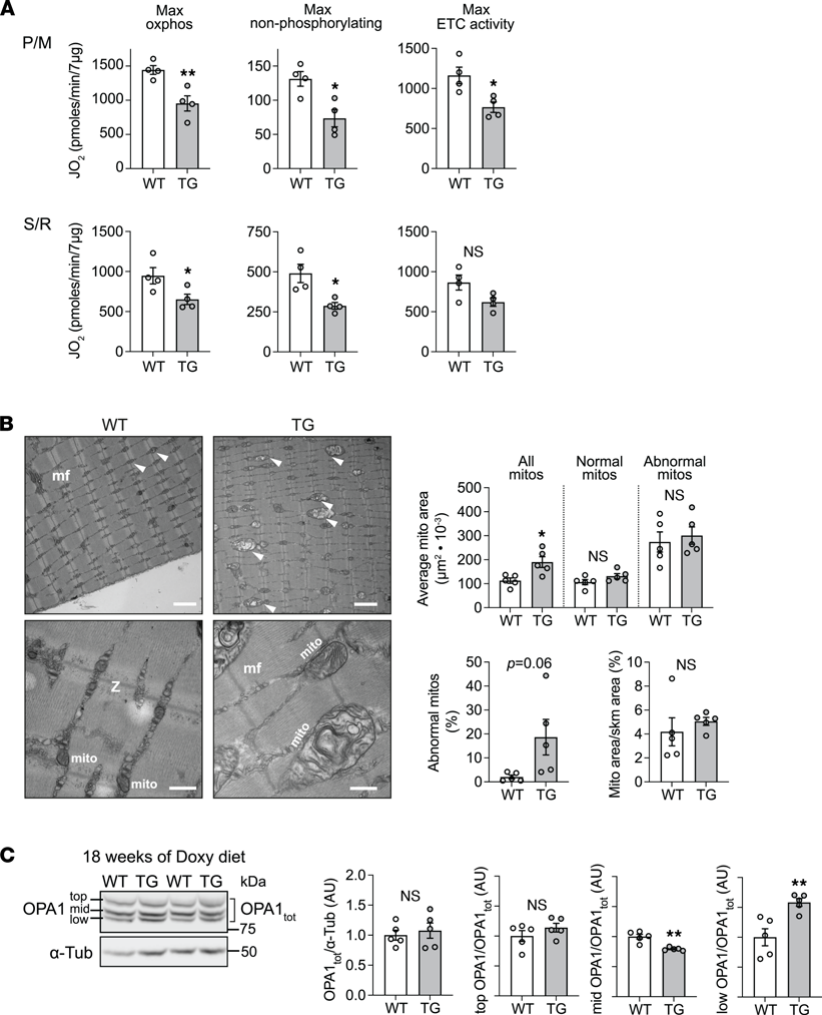
FXN-depleted skeletal muscle exhibits alterations in mitochondrial function and morphology. All measurements were done in samples from WT and TG mice fed with Doxy for 18 weeks. (**A**) Oxygen consumption rate (JO_2_) measured in isolated skeletal muscle mitochondria supplied with pyruvate/malate (P/M; 10 mM/5 mM) or succinate (10 mM + 1 μM rotenone [S/R] to prevent electron backflow through complex I). Max oxphos: JO_2_ reflects maximal oxidative phosphorylation when saturating concentrations of ADP and substrate were used. Max nonphosphorylating: JO_2_ reflects maximal nonphosphorylating oxidation when oligomycin was used to inhibit the ATP synthase. Max ETC activity: JO_2_ reflects the maximal electron transport chain (ETC) activity for the prevailing substrate when the chemical uncoupler FCCP (1 μM) was used. (**B**) *Left*: Representative transmission electron microscopy images from EDL muscle showing mitochondria (arrows: mito), myofibrils (mf), and *z* lines (z). *Upper panels*: Scale bar: 2 μm. *Lower panels*: Scale bar: 500 nm. *Right*: Quantification of mitochondrial (mito) area (average mito area), mito area/skeletal muscle area, and abnormal mitos. Number of fields analyzed: 13–25 fields per sample, 35–125 mitos analyzed per field (total number of mitos per animal: 500–2500). (**C**) *Left*: Representative immunoblots of OPA1 (α-Tubulin: loading control). Major OPA1 forms are depicted by top, middle (mid), lower (low). *Right*: Average values of total OPA1 (OPA1_tot_)/α-Tub, top OPA1/OPA1_tot_, middle OPA1/ OPA1_tot_, and lower OPA1/ OPA1_tot_ (*n* = 5/genotype). All panels: individual data points are shown, and bars represent mean ± SEM. Statistical comparison: unpaired *t* test, *P < 0.05, **P < 0.01.

Impaired mitochondrial function has been shown to activate the ISR in cell and animal models (33–38). We expected that FXN depletion would detrimentally affect oxphos, because several mitochondrial ISC-containing proteins are needed for substrate oxidation required for ATP synthesis. To better contextualize the ISR activation in TG muscle, we wanted to evaluate the extent of oxphos impairment and to determine if other general aspects of mitochondrial health were impaired. Using isolated muscle mitochondria from mice fed with Doxy diet for 18 weeks, we evaluated substrate oxidation as the O_2_ consumption rate (JO_2_), supplying mitochondria with saturating concentrations of pyruvate with malate (P/M) or succinate (with rotenone; S/R). Maximal oxphos (saturating ADP) and nonphosphorylating JO_2_ (“leak,” evaluated in the presence of oligomycin to inhibit the ATP synthase) were significantly lower in TG mitochondria supplied with P/M or S/R (Figure 6A). Maximal ETC capacity (evaluated using the chemical uncoupler FCCP) was significantly lower in TG mitochondria supplied with P/M and trended lower when S/R was supplied (Figure 6A). Thus, as expected, FXN depletion lowered the mitochondrial substrate oxidation capacities.

Using transmission electron microscopy, we observed ultrastructural changes including abnormalities in mitochondria in the EDL muscle from 18-week Doxy-fed mice. In particular, mitochondria in TG muscle were on average larger, and abnormal morphologies (swollen mitochondria, or mitochondria with disrupted cristae structure) were more frequent (Figure 6B; lower right). When mitochondrial size (CSA) was separately considered for normal and abnormal mitochondria, mitochondrial size was similar between WT and TG, for both categories of mitochondria (Figure 6B; upper bar graphs). Mitochondrial density (i.e., the proportion of muscle occupied by mitochondria (normal and abnormal) was also not different between WT and TG (Figure 6B; lower left bar graph). In line with the greater incidence of abnormal mitochondria, we evaluated the processing of OPA1, a dynamin-related GTPase involved in inner mitochondrial membrane fusion and maintaining mitochondrial cristae structure (39, 40). Mitochondrial stress or dysfunction activates OMA1, a metalloproteinase that cleaves OPA1 to generate short forms of OPA1 (41, 42). In cells, ISR activation correlates with OPA1 processing (43, 44). Under FXN depletion, we observed a greater proportion of short forms of OPA1 relative to higher MW forms (Figure 6C), without a significant change in total levels of OPA1 (Figure 6C). These observations indicate that abnormalities beyond an oxphos impairment exist in FXN-depleted muscle mitochondria.

### Exercise training enhances running capacity in FXN-depleted mice but does not alter muscle mass or ISR activation.

**Figure 7:**
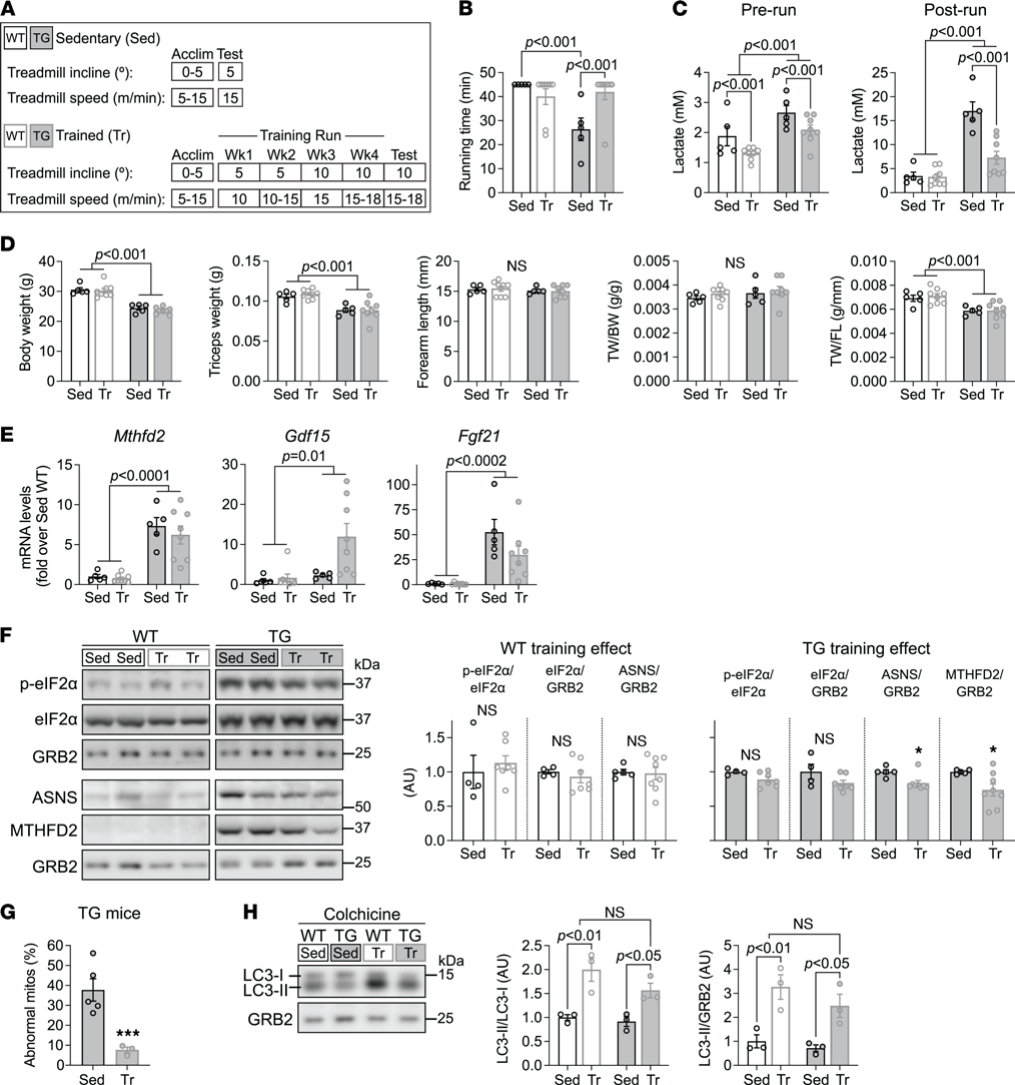
Exercise training enhances running tolerance in FXN-depleted mice but has little effect on the ISR in muscle. All measurements were done in WT and TG mice fed with Doxy for 18 weeks, using quadriceps lysates. (**A**) Treadmill protocol for sedentary (Sed) and trained (Tr) mice. An acclimation period (Acclim) was allowed for both experimental groups. A running test (Test) was performed at the end of the acclimation for Sed mice and at the end of 4 weeks of training for Tr mice. Treadmill incline (°) and speed (m/min) are shown. Speed transitions (e.g., 5–10 and 10–15 m/min) describe initial and final speeds each week or a speed progression on a given day. (**B**) Running time for the Test run (*n* = 5 Sed/8 Tr per genotype). (**C**) Blood lactate levels before and after the Test run (*n* = 5 Sed/8 Tr per genotype). (**D**) BW, TW, FL, TW/BW, and TW/FL, *n* = 5 Sed/8 Tr per genotype. (**E**) Transcript levels (normalized to *Actb*, relative to WT) of *Mthfd2*, *Gdf15*, and *Fgf21* (*n* = 5 Sed/8 Tr per genotype). (**F**) *Left*: Representative immunoblots of p-eIF2α (Ser51), total eIF2α, MTHFD2, ASNS, and GRB2 (loading control). *Right*: Quantification, *n* = 5 Sed/8 Tr per genotype. (**G**) Quantification of abnormal mitochondria (%), by analysis of electron micrographs from quadriceps. Number of fields analyzed: 13–25 fields/sample, *n* = 5 Sed/3 Tr. (**H**) *Left*: Representative immunoblots of LC3 (GRB2: loading control). *Right*: Quantification. Sed and Tr WT and TG mice were treated with colchicine (*n* = 3/group). Quadriceps lysates from Sed mice were also used in Figure 4D. All panels: individual data points are shown, and bars represent mean ± SEM. (**B**–**E** and **H**) Two-way ANOVA, post hoc comparisons: Bonferroni’s correction (*P* values shown). (**F** and **G**) Unpaired *t* test, *P = 0.05, ***P < 0.001.

Daily exercise was shown to delay myopathy progression in the FXN knockin knockout (KIKO) mouse model of FRDA (8). Neither ISR activation nor skeletal mass was evaluated in the KIKO model. Here our goal was to determine whether daily treadmill running would ameliorate or worsen the ISR and skeletal muscle mass in TG mice. After a weeklong acclimation period that started at the beginning of week 12 of Doxy feeding (which is before BW loss in TG mice; ref. 14), mice ran 5 consecutive days per week for 4 weeks (i.e., until the end of week 16 of Doxy feeding). Treadmill speed and incline were gradually increased using a protocol that both WT and TG were able to complete each day (see Figure 7A). We found that TG mice were able to finish a 90-minute running protocol on a flat treadmill, with speed increasing from 5 to 25 m/min (Supplemental Figure 7A), and with little increase in blood lactate (Supplemental Figure 7B). When the treadmill was inclined by 5 degrees, 18-week Doxy-fed untrained WT mice could complete a 45-minute protocol, with the speed increasing from 15 to 18 m/min, whereas 18-week Doxy-fed untrained TG mice could not (Figure 7B), and blood lactate also increased substantially in TG mice (Figure 7C). This protocol was used as the test treadmill protocol conducted during week 17 of Doxy feeding in trained mice, as well as in a group of untrained Doxy-fed littermates. Exercise training improved running capacity in TG mice such that they were able to complete the 45-minute inclined treadmill protocol (Figure 7B). Training also lowered blood lactate levels pre- and postrunning in TG mice (Figure 7C). However, muscle mass, and BW, in trained TG mice were similar to those of sedentary TG mice (Figure 7D). While training lowered the transcript levels of Atrogin-1 (*Fbxo32*) and MuRF-1 (*Trim63*) in TG muscle (Supplemental Figure 8A), training had no effect on ISR activation as determined by levels of p-eIF2α and the gene induction associated with the ISR (Figure 7, E and F), highlighting the major effect of protein translation inhibition over protein degradation in the FXN-depleted muscle, since there were no changes in muscle mass. We did, however, detect slightly lower protein expression of MTHFD2 and ASNS in trained than sedentary TG mice (Figure 7F). We also detected PERK phosphorylation and found no evidence of a gel shift in the PERK signal that would indicate PERK phosphorylation and activation (Supplemental Figure 8B). Additionally, training did not prevent the altered OPA1 processing in the Fxn-depleted muscle (Supplemental Figure 8D) or the lower level of FECH protein in TG muscle (Supplemental Figure 8C). Together, these findings indicate that exercise training was unable to prevent the ISR activation and the lower muscle mass in TG mice.

Finally, the effect of training on the occurrence of abnormal mitochondria was evaluated from electron micrographs of quadriceps from TG mice. The fraction of abnormal mitochondria was substantially lower in muscle from exercise-trained compared with sedentary mice (Figure 7G). This was accompanied by greater LC3-II/LC3-I in muscle from trained versus sedentary TG and WT mice (colchicine-treated) (Figure 7H), indicating greater macroautophagic flux in both WT and TG mice that were exercise trained.

## Discussion

### Summary of results.

Though skeletal muscle has not been considered to be a major source of morbidity in FRDA, it is not neutral to FXN loss. Rather, defective oxphos, correlated with GAA repeat length, is well described in the skeletal muscle of patients with FRDA (4–7). A muscle wasting phenotype (1) and smaller myofiber CSA (10) have also been documented but not quantitatively explored. In adults with FRDA, we found that lean mass indices (*z* scores corresponding to lean body mass and appendicular lean mass indices with and without fat adjustment) were substantially lower compared with healthy individuals with a similar BMI. Children with FRDA also had lower lean body mass compared with a reference population. Importantly, for adults with FRDA, a lower fat-adjusted appendicular lean mass index *z* score was associated with a higher mFARS score and thus with a greater level of dysfunction.

To gain more insight into how skeletal muscle mass could be affected by FXN loss, we took advantage of a mouse model of inducible FXN depletion. The inducible aspect of the model allowed for analysis at different levels of FXN. Mice with approximately 95% loss of FXN, but not approximately 80% loss, exhibited decreased muscle mass gain, with smaller myofiber CSA. Grip strength was also lower in TG mice but maintained the same proportionality to BW as in WT mice, suggesting that lower absolute grip strength in TG mice was fully accounted for by smaller muscle CSA. The decreased muscle mass gain was accompanied by lower global protein translation, with minimal changes in protein degradation and autophagy markers, suggesting that autophagy and degradation were not altered. Consistent with the lower protein translation and lack of increase in autophagy, the ISR was activated whereas AMPK signaling was unchanged. We attempted to determine which of the canonical kinases was responsible for eIF2α phosphorylation and thus ISR activation in TG muscle but could only rule out PERK and GCN2. Finally, though exercise training significantly improved the running capacity of TG mice (Figure 7B), it had no effect on skeletal muscle mass or ISR activation, though there were fewer muscle mitochondria with abnormal ultrastructure.

### Relevance of lean mass score in FRDA.

An obvious implication of lower lean mass for individuals with FRDA is a lower absolute force generation of muscles. But lower lean mass may have further implications. A study focused on bone health in adults with FRDA (45) found substantially lower bone mineral density at skeletal sites affected by mechanical bone loading from movement. Although lean mass was not measured, we suspect that low appendicular lean mass could precede and in part precipitate low bone mineral density found in FRDA.

It is noteworthy that BMI, a clinically available metric of body composition, does not convey important information about lean mass, and lean mass, but not BMI, correlated with disease severity. Similarly, appendicular skeletal muscle mass was a more sensitive predictor of disease severity than BMI in adults with mitochondrial diseases (mitochondrial encephalomyopathy with lactate acidosis, MELAS; and stroke-like episodes and chronic progressive external ophthalmoplegia, CPEO) (46). One previous study in adults also found no difference in BMI and fat mass between patients with FRDA and controls (47), but lean mass variables were not reported. We found that despite having similar overall BMI, lean mass indices in patients with FRDA (adults and children) were substantially lower compared with healthy participants. There are several potential explanations for our findings. First, in adults with FRDA, there was a significant correlation (*P* = 0.02) between ALMI, fat-adjusted ALMI, and lean BMI *z* scores and mFARS scores. As the mFARS contains no elements that primarily reflect strength, this is highly unlikely to be a causal relationship. Although we cannot infer direction of effect, it is possible that individuals who are more affected by FRDA engage in less physical activity and/or exercise, leading to lower ALMI *z* scores. Also, neurological deficits in FRDA may have implications for muscle development and acquisition that remain as of yet unexplored. Lower lean mass in the legs, but not the arms, in adults with FRDA could reflect the effect of loss of ambulation with relative preservation of upper extremity mobility. It is also possible a neurological or developmental cause underlies muscle group differences, but these are incompletely understood. Taken together, our findings suggest that measurements of body composition using DXA may yield important insights in FRDA pathology. Longitudinal assessment would likely further clarify associations between lean mass and disease progression.

### Mechanisms of muscle growth impairment in mice with FXN loss.

Using a mouse model of inducible and progressive FXN loss, we observed lower muscle mass and myofiber CSA in TG mice with approximately 95%, but not 80%, FXN depletion. The temporal aspect of our model allows us to propose that the lower muscle mass reflects impaired muscle mass gain. Lower muscle mass in TG mice was accompanied by less global protein translation. Measures of protein degradation and macroautophagy suggested that these processes were not increased in TG muscle, leaving the suppressed protein translation as the cause of the lower muscle mass. In TG muscle, the changes in major signaling pathways that govern protein translation and disposal are largely consistent with a recalibration of TG muscle toward a smaller mass via lower protein translation. In particular, lower global protein translation is consistent with ISR activation (22) as well as increased levels of total 4E-BP1 that inhibit translation (48). Furthermore, the lack of increase in macroautophagy is consistent with unchanged AMPK signaling. We did observe elevated mRNA for the atrogenes Atrogin-1 and MuRF1 in TG muscle, but the effect, though statistically significant for Atrogin1 (Figure 4B), was not robust, and K-48–linked polyubiquitination of proteins was unchanged. Furthermore, a lower level of p-AKT (Thr308) that could enable atrogene mRNA translation was not detected in TG muscle; rather, p-AKT relative to total AKT was lower because total AKT was elevated. Thus, a deficit in growth factor signaling that would lead to protein degradation does not appear to characterize TG muscle. Yet, we cannot exclude that an anabolic stimulus was blocked in TG muscle.

Regarding the kinase responsible for phosphorylating eIF2α (and thus leading to ISR activation), we were able to rule out PERK and GCN2. Of the remaining 2 canonical kinases, the signal detected using the p-PKR antibody was above the predicted MW of approximately 68 kDa and also above the MW of the positive control; thus, we consider the band in skeletal muscle lysates obtained using the p-PKR antibody to be nonspecific. Evidence for HRI activation was obtained in the heart of TG mice (14) and of the CKM model of FXN loss (49). However, the HRI antibody yielded multiple nonspecific bands in skeletal muscle lysates, preventing a conclusion on the role of HRI in TG muscle. We note, however, that TG skeletal muscle featured a prominent mitochondrial phenotype, characterized by a lower oxphos capacity, the appearance of large mitochondria almost devoid of cristae, and a greater abundance of OPA1 short forms relative to longer forms. OPA1 cleavage of the longest and intermediate-sized forms has been associated with activation of HRI by a soluble fragment of the mitochondrial protein DELE1, generated by the mitochondrial protease OMA1 (43, 44). The heme biosynthetic enzyme, FECH, was also lower in TG muscle, possibly leading to less heme as was found in FXN-depleted mouse liver (50); heme depletion is the canonical activator of HRI (51). At this point we can conclude that, because oxphos deficiency and lower heme levels (inferred from the lower FECH level) can independently drive ISR activation, each condition provides a plausible context for ISR activation in TG muscle.

More generally, though eIF2α phosphorylation has been described in skeletal muscle from mitochondrial myopathy models (34–36, 38) and in denervated mouse muscle (52), the upstream kinase(s) is (are) not well understood in muscle, though some observations have been made. PERK phosphorylation was evident in mouse muscle with partial OPA1 depletion (36), and C2C12 myoblasts and myotubes exposed to mitochondrial toxins responded by the upregulation of ATF4 target genes, with GCN2 serving as the driver in myoblasts, whereas the driver in myotubes was not elucidated (33). The mechanism linking mitochondrial dysfunction to kinase activation is poorly understood beyond the activation of HRI by a soluble fragment of DELE1 generated by the mitochondrial protease OMA1 (and correlated with OPA1 cleavage) that was recently described in cell lines (43, 44). Whether a DELE1-mediated mechanism can also occur in skeletal muscle is unknown, though we have not been able to detect DELE1 (also referred to as Kiaa0141, 0610009O20Rik, or 2700004E22Rik) in proteomics from muscle mitochondria, even after TMT tagging, and even in muscle mitochondria harboring defective oxphos (our unpublished data). Another consideration is ATF4, shown to have a causal role in the aspects of the adaptive response of HeLa cells and human fibroblasts to mitochondrial toxins (37, 53). ATF4 has been considered an important transcription factor in mitochondrial myopathy in mice (34, 35, 38, 53, 54) and humans (55) and in the mouse heart depleted of FXN (14, 49). We could not detect ATF4 at the protein level in muscle lysates or in nuclear fractions. Though problems with antibodies may explain this, the dependence on ATF4 of myopathy adaptation has not been determined. We note that ATF4-dependent phenotypes have been documented in mouse muscle after fasting or limb immobilization (56, 57), demonstrating ATF4’s relevance in skeletal muscle in vivo. Whether ATF3 or ATF5 plays a role in myopathy is also not understood, though ATF5 loss did not affect the response of HeLa cells to mitochondrial dysfunction (37). Yet another consideration is GADD34-mediated negative feedback on p-eIF2α that is considered part of the ISR (30). In that paradigm, GADD34 is upregulated through ISR activation. Yet, we detected only a modest ~25% rise in *Gadd34* mRNA (we could not find a reliable antibody to determine protein levels). Overall, the mechanisms that initiate, maintain, and regulate eIF2α phosphorylation, and how mitochondrial dysfunction initiates the ISR, are major gaps in our understanding of these processes in stressed skeletal muscle and other postmitotic cells.

The ISR was clearly activated on TG muscle, and there was evidence for some increase in mTORC1 signaling. ISR and mTORC1 activation have been described in mouse models of mitochondrial myopathy (34–36, 38, 54). Furthermore, evidence for the activation of one or both of these pathways in the skeletal muscle of humans with mitochondrial myopathy was recently described (55). Thus, it is possible that defective oxphos, or another aspect of mitochondrial dysfunction, in TG muscle leads to the activation of these pathways. Theoretically, this could also occur in some patients with FRDA. ISR activation has also been demonstrated in response to muscle denervation in mice (52), raising the possibility that ISR activation in muscle can have a neurogenic cause. However, evidence does not support a neurogenic cause for ISR activation in TG mice, because regular exercise, which would increase neural input to skeletal muscle, had no effect on ISR activation, and there was no evidence for an additional driver of ISR activation in exercised TG mice. Also, that mass-corrected grip strength was similar in WT and TG mice suggests that muscle innervation was not grossly abnormal in TG mice, though this does not exclude more subtle abnormalities at the neuromuscular junction in these mice. In patients with FRDA, a neurogenic cause for lower lean mass remains a possibility.

Regarding the coactivation of the ISR and mTORC1 pathways, this was also observed in the heart of TG mice (14). Furthermore, as in TG skeletal muscle, TG heart showed smaller myofiber CSA (14). We interpret this to indicate that an mTORC1-driven anabolic stimulus was at least partially blocked by eIF2α phosphorylation. Coactivation of these pathways does not necessarily occur in skeletal myopathy, because muscle depleted of the mitochondrial fission protein Drp1, or OPA1, shows ISR but not mTORC1 activation (34, 35). In general, however, the extent to which these pathways are coactivated in myopathy is not fully appreciated (e.g., refs. 49, 54).

### Mitochondrial phenotype in TG muscle.

Skeletal muscle mitochondria from TG mice with approximately 95% FXN depletion showed lower oxphos capacity. This was not unexpected because FXN is needed for ISC biogenesis, and ISCs are necessary components of several proteins involved in substrate oxidation. Indeed, oxphos defects were documented in the skeletal muscle from patients with FRDA and in skeletal muscle mitochondria from the KIKO mouse model (8). We also analyzed mitochondrial ultrastructure in muscle, which, to our knowledge, has not previously been done in skeletal muscle from patients with FRDA or mouse models. Our analysis revealed mitochondria in TG muscle that were swollen, with cristae disarrangements and a larger average size. FRDA patient–derived lymphoblasts and fibroblasts also display cristae abnormalities (58), and the brown adipose tissue of KIKO mice shows some enlarged mitochondria with disorganized cristae (59). Enlarged mitochondria were also observed in FXN-depleted liver (50). In a more general context of ISC loss, ISCU2-mutant cells had swollen mitochondria with disruption of cristae (60). The mechanisms underlying the changes in ultrastructure remain unexplained. Together, these observations indicate that FXN depletion affects mitochondria beyond the expected decrease in substrate oxidation. In this regard, it is noteworthy that a proteomics analysis of heart mitochondria from TG mice revealed many changes beyond the expected downregulation of ISC-containing proteins (14). How these alterations in mitochondrial structure and function that go beyond an oxphos defect contribute to FRDA pathology requires further study.

### Exercise training.

We determined the impact of regular training on ISR activation and muscle mass in FXN-depleted mice. Our goal with this approach was to challenge TG with a stimulus that would counter any potential effects of low locomotor activity. Additionally, positive effects of exercise on muscle mitochondrial function were described in KIKO mice (8). Exercise training improved running tolerance of TG mice and lowered the levels of blood lactate pre- and postrunning, as reported in KIKO mice (8); thus, exercise training clearly had an impact in TG mice. Aerobic training has also been shown to promote an increase in maximal workload and running capacity in patients with mitochondrial myopathy (61). Yet, exercise training had no effect on muscle mass in TG mice, even though the exercise intervention was started before a BW (and presumably, muscle mass) difference was apparent in TG mice. Exercise had little impact on eIF2α phosphorylation and the downstream transcriptional response. We checked whether exercise-induced ER stress leading to PERK activation could have been a new input that sustained p-eIF2α, but PERK was not activated in trained mice. We did, however, detect small decreases in protein levels of MTHFD2 and ASNS; one possible explanation is that exercise promotes mitophagy and autophagy (62–64). We noted that FECH remained lower in TG muscle following daily exercise, as did the shift in OPA1 from longer to shorter forms. Together, these observations indicate that daily exercise, even starting prior to weight loss, was not able to counter ISR activation and the lower muscle mass in TG mice.

### Study limitations.

The present study does not determine the process within muscle that is responsible for the lower lean mass in individuals with FRDA. More generally, we cannot state that the processes leading to less lean mass in human and mouse muscle are identical. However, that ISR activation has been observed in skeletal muscle under several conditions, including processes initiated by altered innervation (52) and by mitochondrial dysfunction, including in humans (55), supports the possibility of ISR activation in the muscle of individuals with FRDA, at least in those with severe disease.

Additionally, while the muscle phenotype of TG mice correlates with FXN protein expression, a confounding factor is the duration of substantial FXN depletion in muscle. In this regard, it is noteworthy that several mouse models of mitochondrial myopathy, as well as the KIKO model of FRDA, show a progression in phenotype despite stable residual expression of FXN or the other protein being studied; this appears to be the case when the protein is depleted in early life (8, 34, 35, 38, 53) and in adulthood (34, 35). Thus, we cannot determine whether the progressive muscle phenotype in TG mice reflects a further loss in FXN (i.e., from 80% to 95% depleted) or the effect of a prolonged, substantial depletion of FXN. Similarly, it is difficult to separate GAA repeat length and disease duration in individuals with FRDA, since GAA repeat length correlates with the age of disease onset; thus, disease features showing a relationship with GAA repeat length in similarly aged individuals (1, 2) may reflect disease duration. It should be noted that we could not detect a relationship between indices of lean mass and disease severity or GAA repeat length (or disease duration), probably due to insufficient sample size.

In conclusion, these studies in humans with FRDA and mice with progressive FXN depletion suggest that muscle mass maintenance and gain can be compromised in individuals severely affected by FRDA, possibly by ISR activation that is insensitive to aerobic exercise training. To our knowledge, this is the first report of the impact of FXN loss on skeletal muscle mass in a mouse model of FRDA and of stress signaling in skeletal muscle with FXN loss. Whether the ISR is activated in the muscle of patients with FRDA needs future study. The extent to which the ISR activation, and the lack of responsiveness to aerobic exercise, are recapitulated in humans with FRDA also remains to be explored. Candidate interventions to reverse muscle mass deficits, for example resistance exercise training, and/or agents targeting persistent ISR signaling, could also be the focus of future translational investigation; however, the proteostatic changes should be considered adaptive.

## Methods

### Human studies

#### Overview and human patient protocols.

To extend work in animal models, we took advantage of existing body composition data collected during 4 IRB-approved studies in individuals with FRDA, as well as healthy volunteers without FRDA. Demographics, anthropometrics, and DXA scans were abstracted from 3 IRB-approved observational studies at the Children’s Hospital of Philadelphia (CHOP).

#### Participants.

In total, data from 24 adults and 10 children with FRDA, without diabetes, were included. For individuals with FRDA who participated in more than 1 study, the most recent measurement was included (Supplemental Figure 1). Healthy adults (*n* = 24) without FRDA with similar distributions of age, sex, and BMI from 1 of the observational studies were also included.

#### FRDA characteristics.

For individuals with FRDA, FRDA characteristics from ±6 months of the participant’s DXA scan were abstracted from the Friedreich’s Ataxia Clinical Outcomes Measures Study (FACOMS), a multisite, longitudinal, natural history study in which individuals with FRDA participate (65). Time-varying characteristics abstracted included disease duration (measured at the FACOMS visit) and mFARS score, both indices of clinical disease severity (66). Time-invariant characteristics included GAA repeat length on the shorter allele and age of symptom onset, which are both associated with worse disease (67).

#### DXA variables.

LBMI and FMI are variables that reflect the amount of total lean body mass and total fat mass (in kg), indexed to size (height^2^, in m^2^). *Z* scores for LBMI and FMI were calculated for children using a National Health and Nutrition Examination Survey reference cohort (68), while height and BMI *z* scores were calculated using a CDC reference cohort. For adults, *z* scores for ALMI and fat-adjusted ALMI were calculated in addition to FMI and LBMI, relative to population reference cohorts (69). ALMIs were included because they have demonstrated relevance to health and function in adults with other chronic health problems (69).

#### Statistics.

All statistics were performed in Stata, version 16.1 (StataCorp LLC). One- and 2-tailed *t* tests were used to compare normally distributed data between individuals with and without FRDA, while Wilcoxon’s rank-sum tests were used for non-normally distributed data. The Shapiro-Wilk test was used to test for normality. The data from humans are shown as box-and-whisker plots (Figure 1, A and B) or individual values (Figure 1C). A *P* value less than 0.05 was taken to be statistically significant.

### Mouse studies

#### Mouse model.

Mice were maintained at 22°C under a standard 12-hour light/12-hour dark cycle. We used a Doxy-inducible model of FXN knockdown (13, 14). In this model, TG mice contain a Tet-On shRNA (against *Fxn* mRNA) expression cassette. Littermates that do not contain the shRNA cassette were used as controls (WT). Age was matched between WT and TG mice. Starting at 9 weeks of age, Doxy was administered in the chow (200 ppm in Chow 5SHA, Animal Specialties) to WT and TG mice. Only male mice were used for experiments.

Details of the methods used for the mouse studies are available in Supplemental Methods.

#### Statistics.

Data analysis was performed using GraphPad Prism 8.2.1 software. Unpaired 2-tailed *t* test for comparison of 2 means, or 2-way ANOVA followed by Bonferroni’s post hoc comparisons for multiple comparisons, were performed as appropriate. *P* < 0.05 was considered significant. Details specific for a given measurement, including sample sizes, are provided in Results and the figure legends.

### Study approval

Mice were used according to mandated guidelines and protocols of care and use, approved by the Thomas Jefferson University Institutional Animal Care and Use Committee. DXA data were from the following IRB-approved studies at CHOP: IRB 14-011156 (SEM), IRB 14-011655 (DRL), IRB 16-013257 (SEM), and IRB 19-016634 (SEM).

## Author contributions

ELS and SEM conceived the project. CVT, ELS, JD, JIH, BH, JT, MP, DRL, SEM, ZN, and GC designed studies; performed study procedures; and analyzed and interpreted data. SN, AD, KW, and CE performed study procedures. CVT, ELS, JD, JT, and SEM wrote the manuscript. All authors were involved in critical revision of the manuscript.

## Supplementary Material

Supplemental data

## Version 1 05/09/2022

Electronic publication
