# Supplementary material for: Frataxin deficiency lowers lean mass and triggers the integrated stress response in skeletal muscle
Source: JCI Insight. 2022 May 9;7(9):e155201. doi: 10.1172/jci.insight.155201 (PMC9090249; doi:10.1172/jci.insight.155201)

Supplemental Table 1: Summary statistics for adult (without and with FRDA) and pediatric participants (with FRDA).

	Controls (N=24, 12M)	FRDA (N=24, 15M)	Pediatric	FRDA (N=10, 5M)
Sex (%F)	50	37.5	Sex (%F)	50
	**Median (IQR)**	**Median (IQR)**		**Median (IQR)**
Age (Years)	29.1 (24.8, 43.4)	26.5 (23.3, 45.3)	Age (years)	14.1 (12.0, 16.8)
Weight (kg)	75.3 (63.9, 83.0)	67.3 (60.5, 80.4)	Weight (kg)	53.8 (38, 72.9)
Height (cm)	170.4 (163.5, 177.4)	169.6 (166.3, 174.1)	Height (cm)	157.2 (145.4, 167.4)
BMI (kg/m^2)	24.5 (22.1, 27.7)	23.1 (21.2, 28.1)	BMI (kg/m^2)	21.2 (16.6, 26)
GAA repeat length on the least affected allele (base pairs)		533 (300, 700)	GAA repeat length one the least affected allele (base pairs)	833 (766, 900)
mFARS score		48.3 (40, 60.5)	mFARS score	39.2 (32.3, 47.5)
FARS E score		31 (25, 34)	FARS E score	21.2 (17.3, 26)
Age of onset (years)		13 (10, 16)	Age of symptom onset (years)	6.5 (5,8)
Disease Duration (years)		17.0 (12.7, 23.4)	Disease Duration (years)	6.4 (4.4,9)

Demographic and anthropometric data for adults with and without FRDA and children with FRDA (adults: n=24 with and n=24 without FRDA, pediatric n=10). Characteristics of FRDA include GAA repeat length, modified Friedreich's Ataxia Rating Scale (mFARS) scores, FARS E score, age of FRDA symptom onset, and disease duration (adults: n=21 to 24, pediatric n=9 to 10, based on individuals with complete data).

Supplemental Figure 1

A

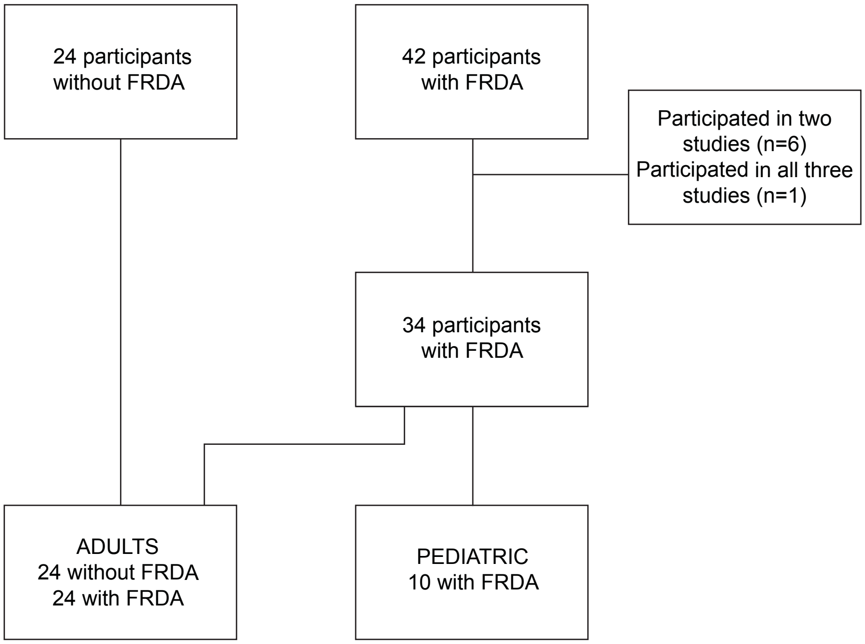

B

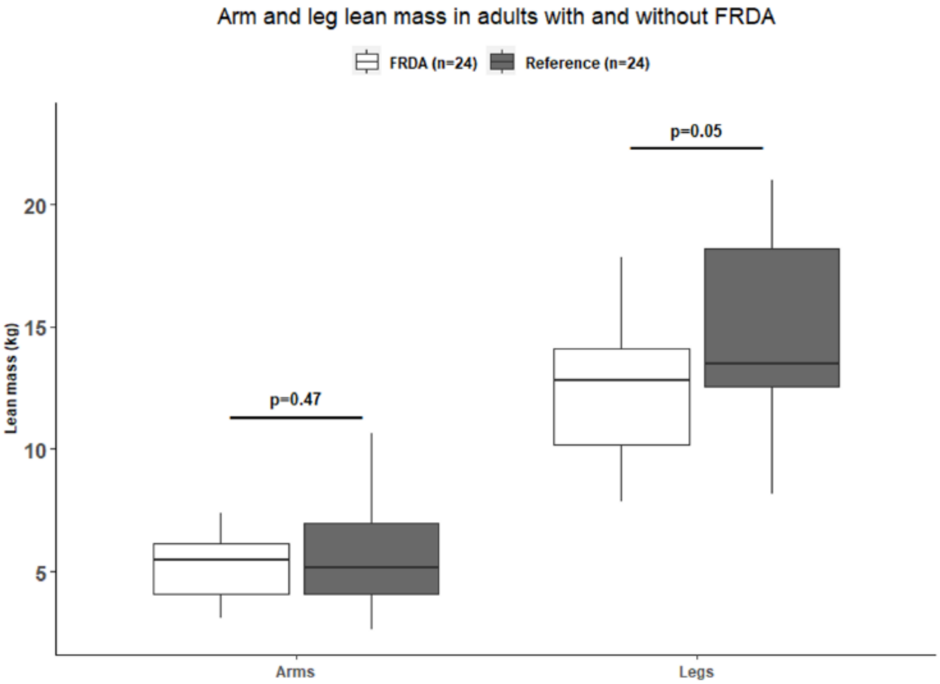

Supplemental Figure 1: Exclusion tree diagram of participants for analysis, and lean mass in arms and legs in adults with FRDA and healthy controls.

(A) Description of how participants were excluded from the analysis. Of the 42 participants, 6 participated in two of the studies, and one participated in all three; in these cases, their most recent scans were analyzed. The remaining 34 participants with FRDA were 24 adults and 10 children. All participants without FRDA were adults.

(B) Total lean mass in bilateral upper extremities and bilateral lower extremities was compared in adults with FRDA and healthy controls using two sample Wilcoxon rank-sum tests. The median values for total leg lean mass were nominally different in adults with FRDA (12.8kg, IQR 10.2-14.1kg) vs. healthy controls (13.5kg, IQR 12.5-18.3kg) (p=0.05). The median values for total arm lean mass were similar between the two groups (FRDA: 5.5, IQR 4.0-6.2 and healthy controls: 5.2, IQR 4.1-7.1) (p=0.47).

Supplemental Figure 2

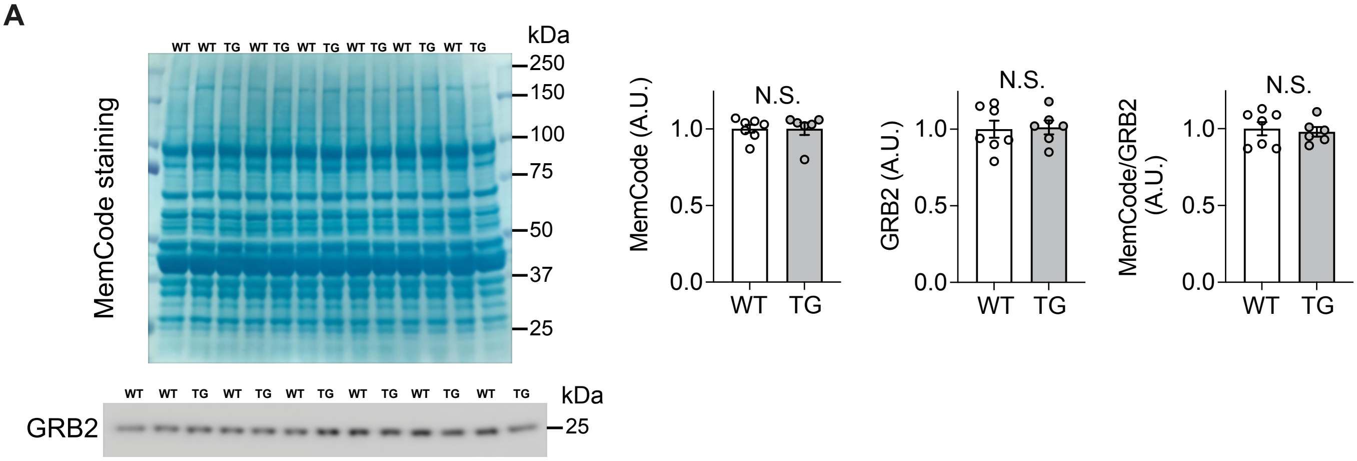

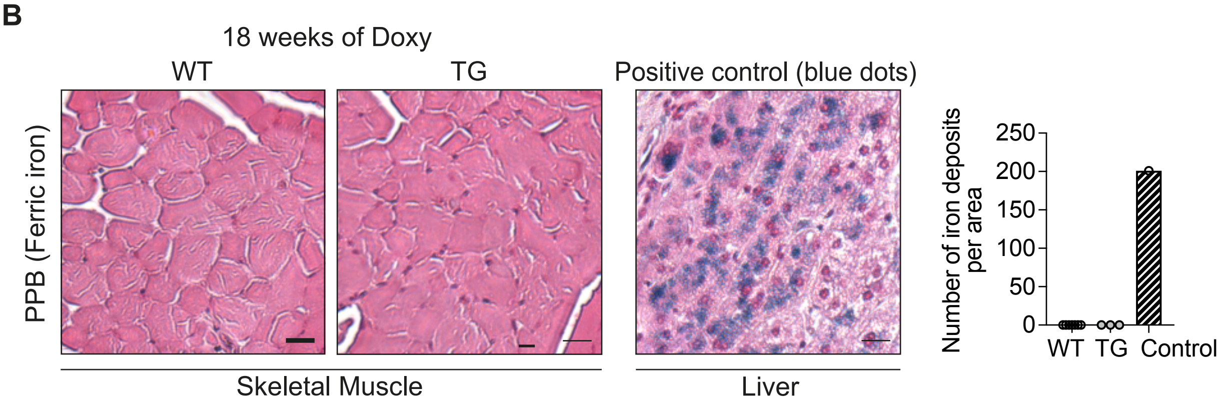

Supplemental Figure 2: Western blot loading control validation, and absence of iron deposition in skeletal muscle from FXN-depleted mice.

(A) *Left*: Representative image of membrane staining (MemCode), used to estimate total protein loading per lane and GRB2, used as a loading control in immunoblots. *Right*: Average values of membrane staining signal (±s.e.m.), GRB2 levels and MemCode/GRB2, along with the individual data points (n=7 WT and 6 TG); N.S.: not significant, unpaired t-test. The membrane used corresponds to Ub-K48 and GRB2 immunoblots, which are shown in Figure 4C.

(B) *Left*: Cross-sections of EDL muscles from WT and TG mice fed with Doxy diet for 18 weeks, stained with Perls Prussian Blue (PPB) to detect iron deposition (as ferric iron). A human liver sample (hemochromatosis) was used as a positive control for PPB staining. *Right*: Quantification of iron deposits per area (353 μm x 264 μm) for skeletal muscle from WT and TG (n=7 WT and 3 TG) and positive control (human liver sample). Values are mean ± s.e.m (except for the positive control, which is n=1).

Values are mean ± s.e.m. Statistical comparison was by unpaired t-test, N.S.: not significant.

Supplemental Figure 3

A

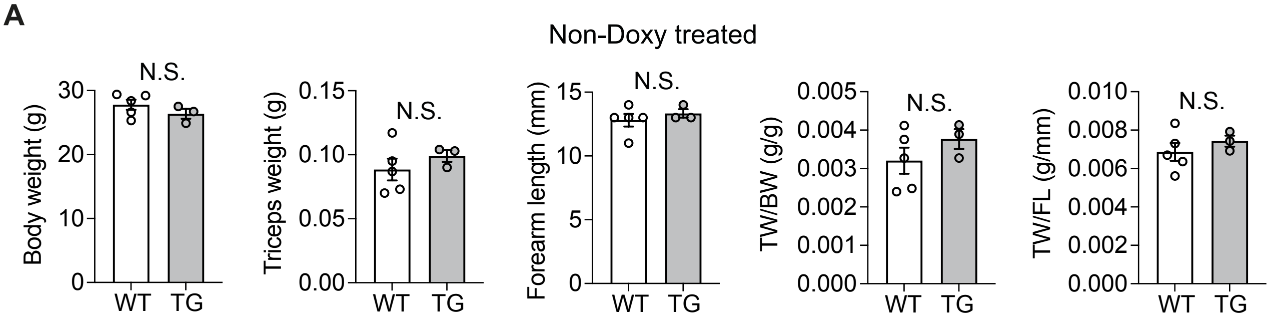

B

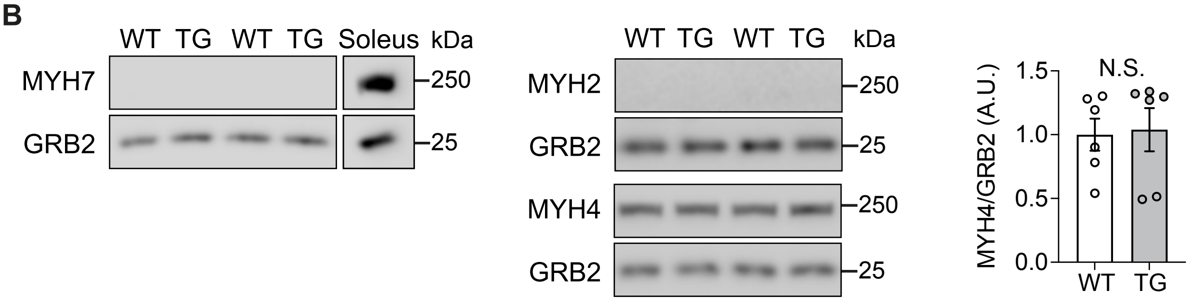

Supplemental Figure 3: Morphometric parameters in non-Doxy treated TG mice, and fiber type evaluation.

(A) Body weight (BW), triceps weight (TW), forearm length (FL), triceps weight to BW ratio (TW/BW) and TW to forearm length ratio (TW/FL) comparison between WT and TG mice fed with regular chow (i.e., not fed with the Doxy diet) (n=5 WT/3 TG). Values are mean ± s.e.m. Statistical comparison was by unpaired t-test, N.S.: not significant (unpaired t-test).

(B) *Left***:** Representative immunoblots of MYH7 (type I muscle fiber), MYH2 (type IIa) and MYH4 (type IIb). GRB2 was used as a loading control. For MYH7 detection, a positive control for type I fibers (soleus) was included. Bar chart shows means±s.e.m as well as individual data points for each sample, n=6/genotype, N.S.: not significant (unpaired t-test).

Supplemental Figure 4

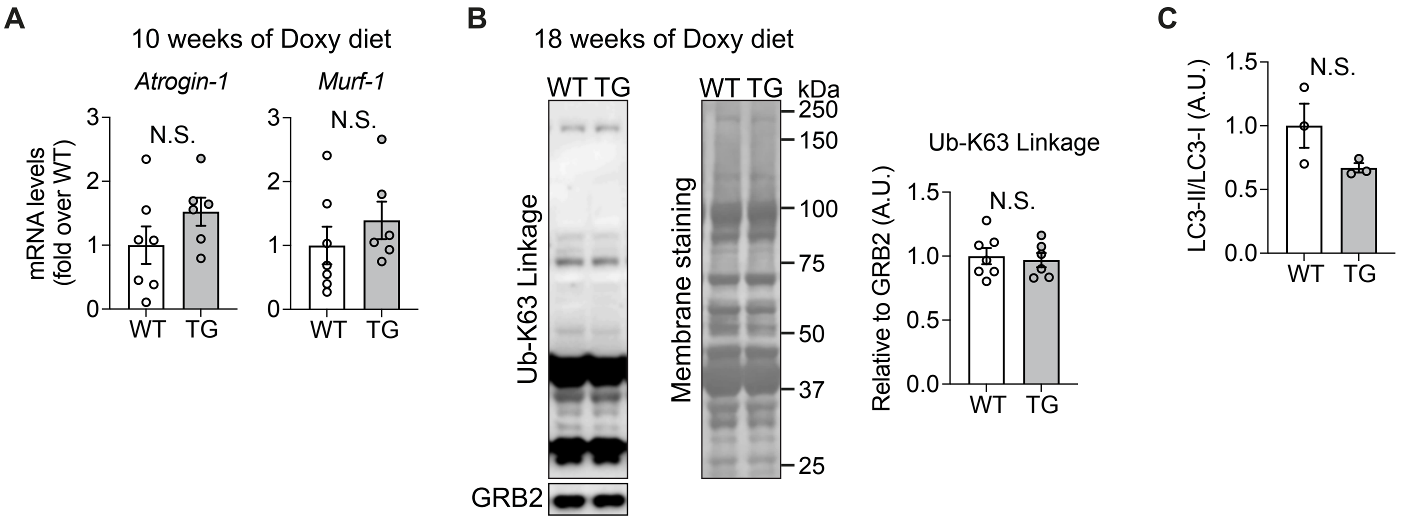

Supplemental Figure 4: Transcript levels of atrogenes in quadriceps of 10 weeks Doxy-fed mice and K63-linked ubiquitination (Ub-K63) in quadriceps of 18 weeks Doxy-fed mice.

(A) Transcript levels (normalized to *Actb* (β-actin*)* and expressed relative to WT) of *Atrogin-1* and *MuRF-1* (n=7 WT/6 TG).

(B) *Left***:** Representative immunoblots of Ubiquitin (Ub) and K63-linked ubiquitination (Ub-K63) from quadriceps lysates from 18 weeks Doxy-fed mice. GRB2 was used as loading control. Membrane staining (MemCode) was used to estimate protein loading. *Right*: Averaged values of Ub-K63/GRB2 (n=7 WT/6 TG).

(C) Quantification of LC3-II/LC3-I (n=3/genotype) from WT and TG mice treated with colchicine (0.4 mg/kg).

In all panels, bars represent mean ± s.e.m. Statistical comparison was by unpaired t-test, N.S.: not significant.

Supplemental Figure 5

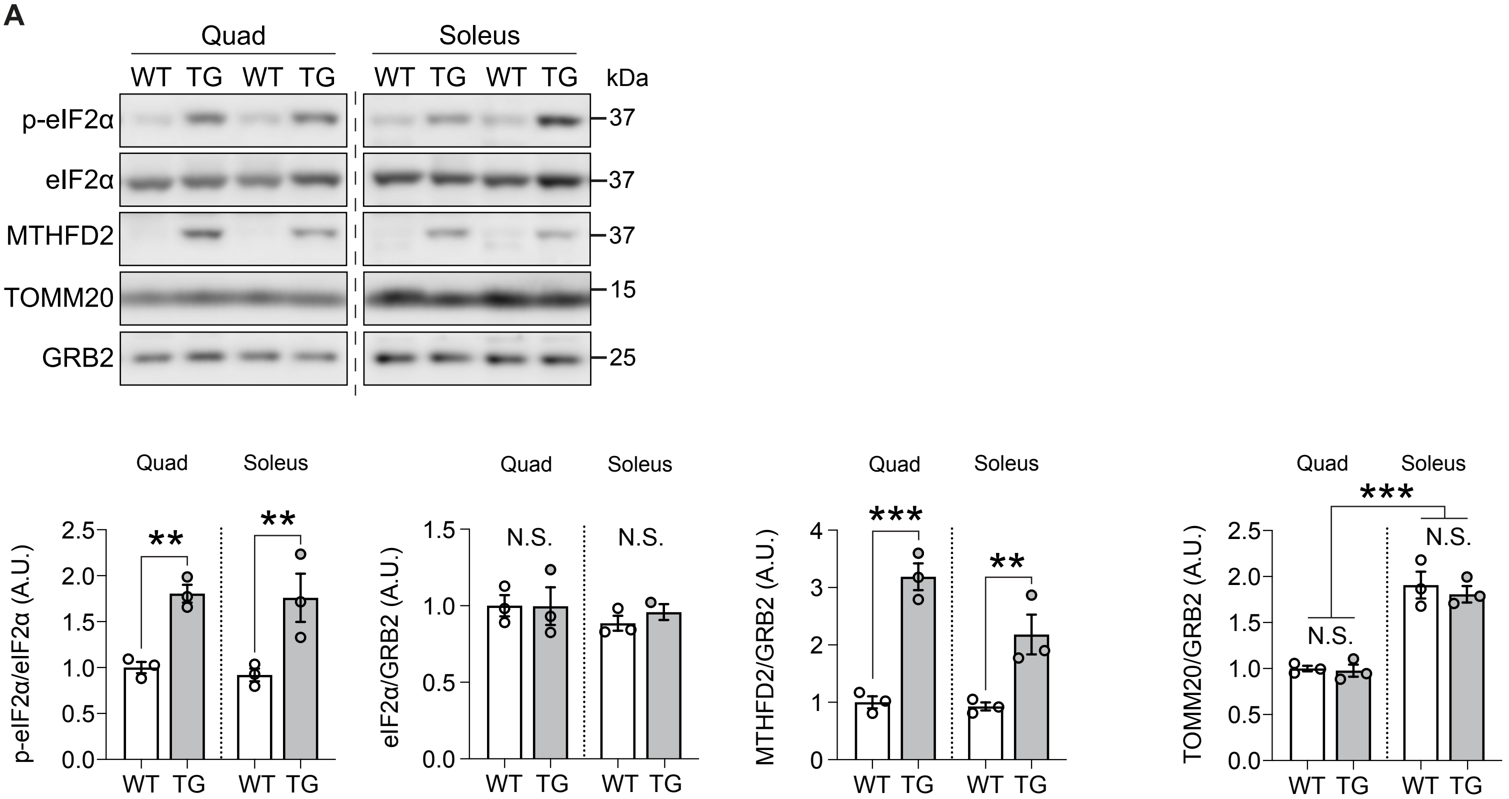

Supplemental Figure 5: Integrated stress response is evident in Soleus from TG mice

(A) *Top:* Representative immunoblots of p-eIF2α (Ser51), total eIF2α, MTHFD2, and TOMM20 (as a surrogate for mitochondrial content) from Quadriceps (quad) and Soleus lysates. GRB2 was used as the loading control. *Bottom:* Averaged values of p-eIF2α/eIF2α, eIF2α/GRB2, MTHFD2/GRB2, and TOMM20/GRB2 (n=3/genotype).

All panels: Individual data points are shown, and values are mean ± s.e.m. Statistical comparison was by unpaired t-test (p-eIF2α, eIF2α, MTHFD2), or two-way ANOVA and post-hoc comparisons using a Bonferroni correction (p values) for TOMM20. P values <0.05 were considered significant. N.S.: not significant.

Supplemental Figure 6

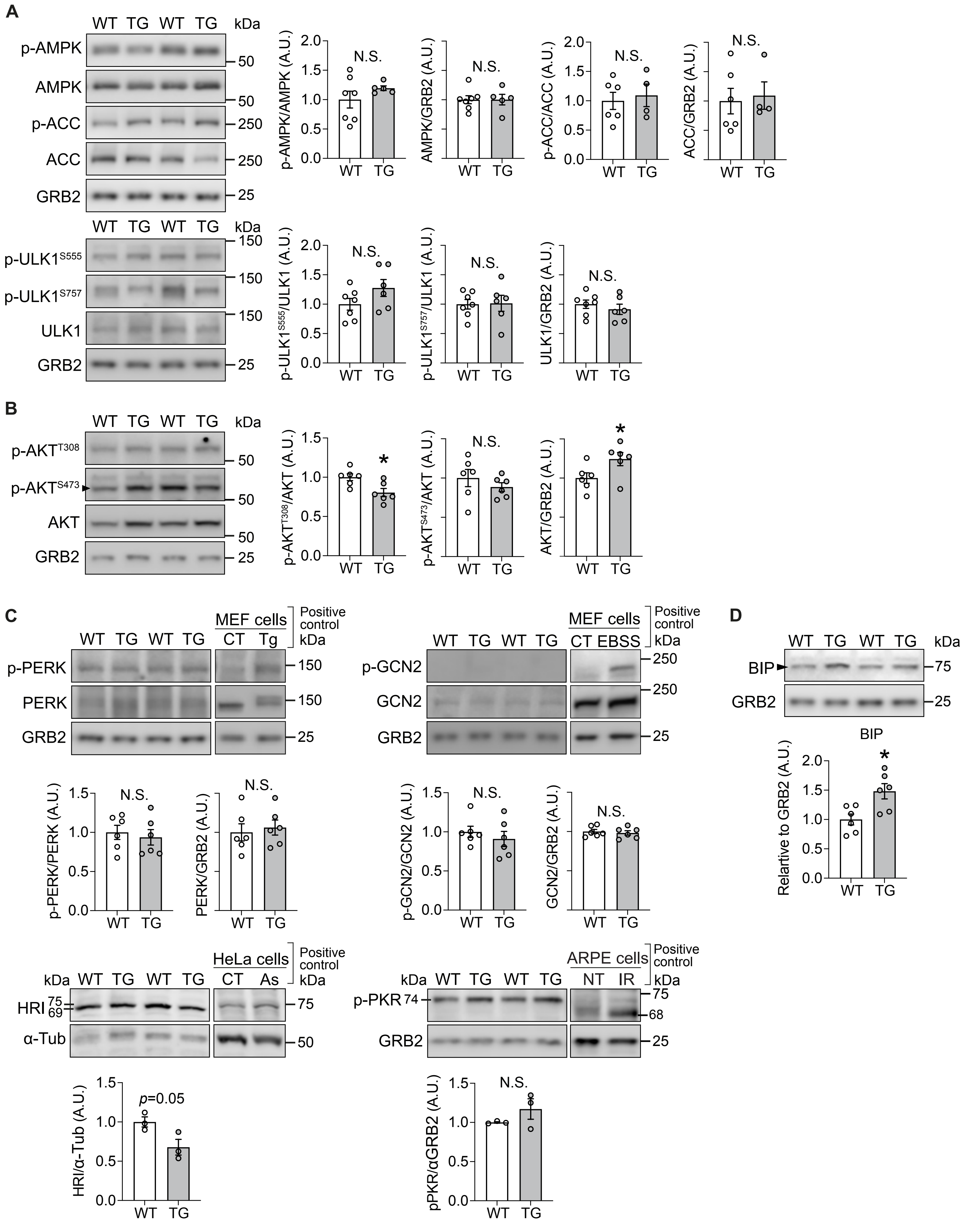

Supplemental Figure 6: Absence of change in AMPK or AKT signaling, and lack of PERK and GCN2 activation, in quadriceps from FXN-depleted mice.

(A) *Top:* Representative immunoblots of p-AMPK (Thr172), total AMPK, p-ACC (Ser79) and total ACC (GRB2: loading control). *Bottom*: Representative immunoblots of p-ULK1 (Ser555), p-ULK1 (Ser757) and total ULK1 (GRB2: loading control). Averaged values of p-AMPK/AMPK, AMPK/GRB2, p-ACC/ACC, ACC/GRB2 (n=6-7 WT/4-5 TG) and p-ULK1(Ser555)/ULK1, p-ULK1(Ser757)/ULK1 and ULK1 (n=7 WT/6 TG) are shown on the right of the corresponding immunoblot set.

(B) *Left*: Representative immunoblots of p-AKT (Thr308), p-AKT (Ser473) and total AKT (GRB2: loading control). *Right*: Averaged values of p-AKT(Thr308)/AKT, p-AKT(Ser473)/AKT and AKT/GRB2 (n=6 per genotype).

(C) Representative immunoblots of p-PERK (Thr980), total PERK, p-GCN2 (Thr899), total GCN2, HRI and p-PKR (Thr451), with GRB2 and α-Tubulin (α-Tub) as loading controls. Averaged p-PERK/PERK, p-GCN2/GCN2, HRI/(α-Tub) and pPKR/GRB2 values are shown below respective immunoblot, (n=3-6/genotype). Positive controls for antibodies: p-PERK: MEFs cells treated with Thapsigargin 1 μM (Tg) for 1 hr to generate ER stress; p-GCN2: MEF cells cultured in EBSS for 1 hr to induce amino acid deprivation; p-PKR: ARPE-19 cells were irradiated (20gy) to cause DNA damage (NT: non-treated, IR: irradiated); HRI: HeLa cells were incubated with 100 μM Na-Arsenite (As) for 1 hr.

(D) *Top*: Representative immunoblots of BIP and GRB2 (loading control). *Bottom*: Averaged values of BIP/GRB2 (n=6-7/genotype).

In all panels, individual data points are shown, and bars represent mean ± s.e.m. Statistical comparison was by unpaired t-test, *$p<0.05$, N.S.: not significant.

Supplemental Figure 7

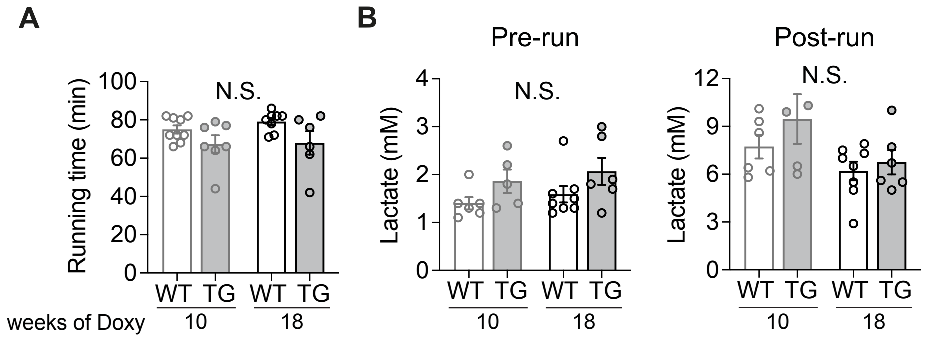

Supplemental Figure 7: No difference between WT and TG mice in running time or in lactate levels when mice were challenged with a flat treadmill running protocol.

(A) Running time values for mice fed with Doxy for 10 weeks (n=9 WT/7 TG) and 18 weeks (n=8 WT/6 TG).

(B) Blood lactate levels pre- and post-running for WT and TG mice fed with Doxy diet for 10 weeks (n=5/genotype) and 18 weeks (n= 8 WT/6 TG).

All panels: Individual data points are shown, and values are mean ± s.e.m. Statistical comparison was by two-way ANOVA and post-hoc comparisons using a Bonferroni correction (p values). P values <0.05 were considered significant. N.S.: not significant.

Supplemental Figure 8

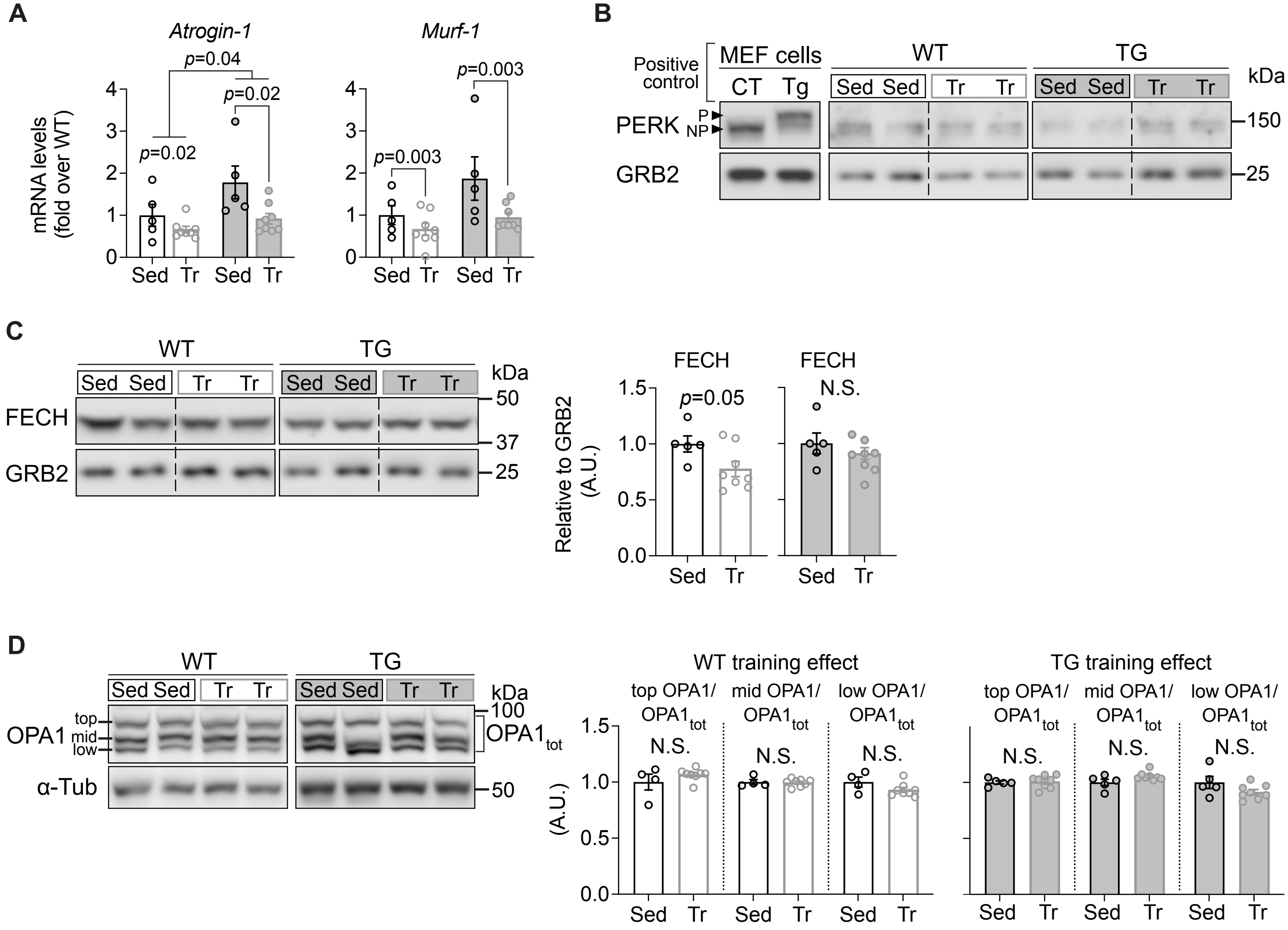

Supplemental Figure 8: Lack of effect of training on transcript levels of atrogenes, PERK and FECH protein levels.

(A) Transcript levels (normalized to *Actb* (β-actin*)* and expressed relative to WT) of *Atrogin-1* and *MuRF-1* for sedentary (Sed) and Trained (Tr) WT and TG mice, (n=5 WT/8 TG).

(B) Representative immunoblots of PERK and GRB2 (loading control) for sedentary (Sed) and Trained (Tr) WT and TG mice (n= 5-7 Sed/8 Tr per genotype). A positive control for PERK signal gel shift (indicative of phosphorylation) was generated using MEFs cells treated with Thapsigargin 1 μM (Tg) for 1h. P = Phosphorylated, NP = Non-Phosphorylated.

(C) Representative immunoblots of FECH and GRB2 (loading control) for sedentary (Sed) and Trained (Tr) WT and TG mice (n= 5 Sed/8 Tr per genotype).

(D) *Left*: Representative immunoblots of OPA1 (α-Tubulin: loading control). Major OPA1 forms are depicted by top, middle (mid), lower (low) in the membrane. *Right*: Averaged values (± s.e.m.) of total OPA1 ($OPA1_{tot}$)/α-Tub, top OPA1/ $OPA1_{tot}$, middle OPA1/ $OPA1_{tot}$ and lower OPA1/ $OPA1_{tot}$, (n=5 Sed/8 Tr per genotype). N.S.: not significant, unpaired t-test.

Supplemental Materials and Methods

Mouse studies

Grip strength test. Grip strength was measured using the DFIS-2 Series Digital Force Gauge (Columbus Instruments, OH), as described (70). Grip strength testing was conducted by allowing the mouse to grasp a triangular metal bar that is attached to the force gauge. During the testing session, the mouse was allowed to grab the metal bar with both forepaws and then was quickly pulled away from gauge instrument, until the forelimbs released the bar. This provides a measurement of the force of grip strength. A similar procedure was used using mouse hindlimbs. During each test, six measurements were recorded for each forelimb and hindlimb, using an interval of at least 60 s between each measurement. Mice were acclimated for two weeks (with two acclimation sessions per week) before the testing session.

Treadmill training protocol. Treadmill running was performed using an Exer3/6 Treadmill (Columbus Instruments). For the inclined treadmill training protocol (see Fig. 7A), WT and TG mice were separated in two experimental groups, sedentary and trained. Trained mice underwent an acclimation period of 4 days on the treadmill, before the training phase of four weeks. During the first of training week, the treadmill was set at 5° of incline and 10 m/min of speed. The second week consisted of a 5° incline and 10 -15 m/min of speed. At the third week, treadmill incline was 5° and speed was 15 m/min. The last week of training consisted of 10° of incline and 15-18 m/min speed. After the training phase, a running test was performed under similar conditions (10° incline and 15-18 m/min speed). The total running time for the training and running test was 45 min. Lactate levels were measured with a Lactate Plus Meter (Nova Biomedical) at rest (pre-run) and after the mouse had stopped running (post-run). The sedentary group was acclimated before an abridged running test (5° incline and 15 m/min speed), without any training. The shock grip (20V) was turned on during acclimation, training and running test.

For the flat treadmill protocol, WT and TG were acclimated for 3 days. An experimental run was performed two days later, where the treadmill speed was increased progressively. The speed was increased from 0 m/min to 5 m/min (20 s of speed transition) and was maintained at 5 m/min for 720 s. From here, the speed was increased subsequently (60 s of transition for each speed) to 7.5 m/min (duration of 720 s) and then to 10 m/min (for 720 s). After this phase, the speed was progressively increased (60 s of transition for each speed) to 12.5, 15 and 17.5 m/min (600 s of

duration for each period). Then, the treadmill speed was increased (60 s of transition) to 20 and 22.5 m/min (duration of 360 s for each speed). Finally, the speed was increased to 25 m/min (60 s of transition) and was maintained at this speed for 240s. The total running time was 90 min.

Isolation of skeletal muscle mitochondria. Skeletal muscle mitochondria were isolated following the protocol described in (71). All steps were performed at 4°C or on ice. Skeletal muscle from mouse forelimb and hindlimb was rapidly dissected and placed in basic medium (BM: 140 mM KCl, 20 mM HEPES, 5 mM $MgCl_2$, 1 mM EGTA, pH 7.0). Muscle was cleaned of fat and connective tissue, minced with a sharp blade, and placed in 30 mL of homogenization medium (HM: BM with 1 mM Mg-ATP, 2 mM EGTA and 1% BSA (w/v)) containing two units of protease from Bacillus licheniformis (Sigma P5380) per muscle weight (g)). The tissue was homogenized using a glass Potter-Elvehjem homogenizer with a Teflon pestle (500 r.p.m., 15 passes). After that, the tissue suspension was centrifuged at 2,000 r.p.m. for 10 min and the supernatant was collected and spun at 9,000 r.p.m. for 8 min. The pellet obtained was resuspended and incubated in BM for 5 min. Samples were then centrifuged at 2,000 r.p.m. for 10 min and supernatant obtained was filtered through a 70 μM cell strainer (Falcon 352250) and spun at 10,000 r.p.m. Finally, the pellet was resuspended in BM and its protein concentration was determined by bicinchoninic acid (BCA) assay (ThermoFisher Scientific 23225).

Bioenergetics analyses in isolated skeletal mitochondria. O_2 consumption (JO_2) was measured using the Seahorse XF24 Analyzer (Seahorse Bioscience, Billerica, MA, USA). Isolated mitochondria were studied essentially as we have done previously (13). Each well of the custom microplate contained 7 μg of mitochondria suspended in mitochondria assay medium (MAS; 70 mM sucrose, 22 mM mannitol, 10 mM KH_2PO_4, 5 mM $MgCl_2$, 2 mM HEPES, 1 mM EGTA, 0.2% defatted BSA, pH 7.4 at 37°C). The amount of mitochondria used was optimized to obtain a linear O_2 *vs*. time signal under all conditions. The microplate was centrifuged at 2,000 *g* for 20 min at C, to promote adhesion of mitochondria to the plastic. Different substrates were tested: malate-pyruvate (5 mM/10 mM) and succinate (10 mM + 1 μM rotenone to inhibit Complex I and thereby prevent reverse electron flow through that complex). ADP (2.8 mM) was used to measure maximal phosphorylating (max oxphos) JO_2, oligomycin (4 μg/mL) was used to measure maximal non-phosphorylation "leak" respiration JO_2 and the uncoupler FCCP (6.7 μM) was used to measure maximal electron transport chain (ETC) activity.

Immunoblot analysis. For western blot analysis using mouse tissue, quadriceps were quickly frozen in liquid nitrogen. Then, tissue was homogenized over ice in a lysis buffer containing: 150 mM NaCl, 25 mM HEPES, 2.5 mM EGTA, 1% Triton 100X, 1% Igepal (10%), 0.10% SDS, 0.1% Deoxycholate, 10% Glycerol, protease inhibitor (Roche 11873580001) and phosphatase inhibitor cocktail (200 mM sodium fluoride, 200 mM, imidazole, 115 mM sodium molybdate, 200 mM sodium orthovanadate, 400 mM sodium tartrate dihydrate, 100 mM sodium pyrophosphate and 100 mM β-glycerophosphate), using a glass/Teflon homogenizer at 500 rpm. After that, quadriceps lysates were incubated at 4°C for 45 min, then centrifuged at 18,000 *g* for 20 min. Protein concentration was measured from the supernatant by BCA assay. For western blot analysis from cultured cells, cells were lysed in RIPA buffer (Sigma-Aldrich R0278) containing protease and phosphatase inhibitors (as above). Cell lysates were centrifuged at 12,000 r.p.m. for 10 min. From the supernatant, protein concentration was measured by BCA assay. For LC3 detection in skeletal muscle, we used T-PER tissue protein extraction reagent (ThermoFisher Scientific 78510). Quadriceps were quickly pulverized over dry ice, and then incubated for 1h in T-PER at 4°C (shaking them at 1,500 r.p.m.). After that, samples were centrifuged at 18,000 *g* for 30 min at 4°C. Supernatant protein concentration was measured by BCA assay. Primary antibodies were used for overnight incubation diluted in TBS-T 1% (20 mM Tris, 0.9 % NaCl, 0.1 % Tween 20, pH 7.4) and are listed below.

Primary Antibodies used in immunoblot analysis

**Antibody**	**Catalog Number**	**Dilution**	**Host**
Frataxin (FXN)	Abcam (Ab)175402	1:500	Rabbit
UQCRC2 (C-III)	Ab14745	1:1000	Mouse
Transferrin receptor (TFR)	Thermofisher Scietific H68.4	1:500	Mouse
Ferroportin/FPN1/SLC40A1	Novus Biological NBP1-21502SS	1:1000	Rabbit
FTH	Cell Signaling Technology (CST)-3998	1:1000	Rabbit
Ferrochelatase (FECH)	SC-377377	1:1000	Rabbit
GRB2	SC-8034	1:1000	Rabbit
Anti-Puromycin	Millipore Sigma MABE343	1:5000	Mouse
Ubiquitin	CST-3936	1:1000	Mouse
K48-linkage specific Polyubiquitin	CST-8081	1:1000	Rabbit

K63-linkage specific Polyubiquitin	CST-5621	1:1000	Rabbit
LC3B	CST-3868T	1:1000	Rabbit
SQSTM1/p62	CST-5114S	1:1000	Rabbit
Phospho-eIF2α (Ser51)	CST-3398	1:1000	Rabbit
eIF2α	CST-5324	1:1000	Rabbit
Phospho-P70-S6K (Thr389)	CST-9234	1:1000	Rabbit
P70-S6K	CST-9202	1:1000	Rabbit
Phospho-S6 (Ser235/236)	CST-2211	1:1000	Rabbit
S6	CST-2217	1:1000	Rabbit
Phospho-4E-BP1 (Thr70)	CST-9455	1:1000	Rabbit
4E-BP1	CST-9644	1:1000	Rabbit
Phospho-AMPK (Thr172)	CST-2535	1:1000	Rabbit
AMPKα	CST-2532	1:1000	Rabbit
MTHFD2	Proteintech 12270-1-AP	1:1000	Rabbit
ASNS	Proteintech 14681-1-AP	1:1000	Rabbit
Phospho-ACC (Ser79)	CST-3661L	1:1000	Rabbit
ACC	CST-3676T	1:1000	Rabbit
ULK1	CST-8054T	1:1000	Rabbit
Phospho-ULK1 (Ser757)	CST-6888T	1:1000	Rabbit
Phospho-ULK1 (Ser555)	CST-5869T	1:1000	Rabbit
Phospho-AKT (Thr308) XP	CST-13038	1:1000	Rabbit
Phospho-AKT (Ser473) XP	CST-4060	1:1000	Rabbit
AKT	CST-9272	1:1000	Rabbit
Phospho-GCN2 (Thr899)	Ab75836	1:1000	Rabbit
GCN2	CST-3301S	1:1000	Rabbit
Phospho-PERK (Thr980)	CST-3179	1:1000	Rabbit
PERK	CST-5683	1:1000	Rabbit
Phospho-PKR (Thr451)	Invitrogen 44-668G	1:1000	Rabbit
HRI	EMD Millipore 07-728	1:5000	Rabbit
α-Tubulin	CST-2125S	1:1000	Rabbit
OPA1	BD Transduction Laboratories	1:1000	Mouse
BIP	CST-3177	1:1000	Rabbit

Cell culture and stress conditions for antibody positive controls.

MEF and HeLa cells were used to generate positive control conditions to validate immunoblot detection of ISR kinase (PERK, GCN2, HRI and PKR) activation by commercial antibodies. MEF and HeLa cells were cultured in Dulbecco's modified Eagle's medium (11965-118, Invitrogen) supplemented with 10% fetal bovine serum, 2 mM glutamine and 100 U/ml penicillin and

100mg/ml streptomycin (ThermoFisher Scientific 10378016), and maintained in a 5% CO_2-humidified air at 37°C.

To validate p-PERK antibody, MEF cells were treated with thapsigargin (Tg 1µM) for 3 h to induce ER stress. For the p-GCN2 antibody, MEF cells were incubated in Earle's Balanced Salt Solution (EBSS; 5.33 mM KCl, 26.19 mM $NaHCO_3$, 117.24 mM NaCl, 1.104 mM NaH_2PO_4 and 5.56 mM D-Glucose, pH 7.4) for 3 h, to generate an amino acid deprivation condition. A positive control for HRI activation was generated using HeLa cells treated with sodium arsenite (100µM $AsNaO_2$) for 1 h (72). Finally, for the p-PKR antibody, ARPE-19 cells (human retinal pigment epithelium cells) were irradiated with a CellRad350 at 0.8-0.9 Grey per minute.

Quantitative polymerase chain reaction. Mouse quadriceps were used to extract total RNA using Animal Tissue RNA Purification Kit (Norgen Biotek Corp, 25700), following the manufacturer's instructions. RNA concentration was measured by Nanodrop One (ThermoFisher Scientific). RNA was reverse transcribed using oligo(dT) primers and SuperScript III (Invitrogen, 18080-051). Primers were checked for specificity and efficiency. qPCR reactions were performed using SYBR™ (Invitrogen, 4472908), with 20 ng cDNA/reactions, using a QuantStudio™ 5 Real-Time PCR Instrument (Applied Biosystems). The ΔΔCt method was used to calculate mRNA levels relative to *Actb* (β-actin). Primers sequences used are listed below.

Primers for qPCR (all are mouse-specific)

Primer name		Sequence
Fbxo32 (Atrogin-1)	Forward	ACCTGCTGGTGGAAAACATC
	Reverse	CTTCGTGTTCCTTGCACATC
Trim63 (MuRF-1)	Forward	AAGGAGCGCCATGGGATACTG
	Reverse	GTCACGCACGATTTCCC
Mthfd2	Forward	AGGTCCCAAGCCTTTGAGTT
	Reverse	GTAAGGGAGTGCCGTTGAAA
Slc7a5	Forward	TGCAGCCATGACCCTAACAG
	Reverse	AACAATGGGGACAGACCAGG
Asns	Forward	TGGCTGCCTTTTATCAGGGG
	Reverse	CAGATGCCCGAACTGTCGTA
Fgf21	Forward	ATGGAATGGATGAGATCTAGAGTTGG
	Reverse	TCTTGGTGGTCATCTGTGTAGAGG
Gdf15	Forward	GAGCTACGGGGTCGCTTC
	Reverse	GGGACCCCAATCTCACCT
Ppp1r15a (Gadd34)	Forward	GGCGGCTCAGATTGTTCAAAGC
	Reverse	CCAGACAGCAAGGAAATGGACTG

Actb (β-actin)	Forward	CAACACCCCAGCCATG
	Reverse	GTCACGCACGATTTCCC

Global protein synthesis (SUnSET). To estimate global protein synthesis, we used an *in vivo* SUnSET assay (18). A sterilized puromycin (Sigma, 8833) solution (in PBS) was injected intraperitoneally (I.P.). Each mouse received 0.04 μmol/g. Thirty minutes after the injection, animals were sacrificed to harvest quadriceps and tibialis anterior (TA) muscle for western blot detection of incorporated puromycin into nascent peptides or proteins.

Colchicine administration. To evaluate the levels of lipidated LC3-II in skeletal muscle, a colchicine (Millipore-Sigma C9754) solution (in PBS) was in injected intraperitoneally (I.P.). Each mouse received 0.4 mg/kg per injection. A second injection was administered after 12 h after the first injection. After 12 h of the second injection, mice were sacrificed to harvest quadriceps for western blot detection of LC3.

Histology. For histological analysis, EDL (extensor digitorum longus) muscles were quickly dissected, pinned onto a silicon surface (to prevent muscle shrinkage) and fixed in 4% paraformaldehyde and then washed and incubated in PBS. Hematoxylin/Eosin (H&E) and Perls Prussian Blue (PPB) staining were performed at the Translational Research/Pathology shared resource at Thomas Jefferson University. An Olympus CKX41 inverted microscope and cellSens Standard software were used for image acquisition. Images were analyzed using Image J software. For H&E, the cross-sectional area (CSA) of ~ 25 individual muscle fibers was measured per image (six 10x digital images per sample). For PPB staining, five 20x digital images from each tissue section were analyzed. Images were converted to an RGB stack and PPB positive particles were counted using Particle Analyzer plugin, establishing a threshold in the red channel. Only particles of area ≥ 5 μm^2 were considered for the quantification. The size of the area analyzed was 353 μm x 264 μm (2048 x 1536 pixels). A PPB staining positive control (human hemochromatosis liver sample) was provided by the Translational Research/Pathology shared resource at Thomas Jefferson University.

Transmission electron microscopy. EDL muscles were quickly isolated and pinned onto coverslips coated with a silicon surface and fixed with 6% glutaraldehyde in 0.1 M sodium cacodylate buffer for 1 hour (EMS Science – Hatfield, PA, USA). The muscles were cut into small

pieces (~1 mm^3) and fixed for an additional 10 min in 6% glutaraldehyde in 0.1 M sodium cacodylate buffer, and overnight postfixed in 4°C in 2% osmium tetroxide (EMS Science) partially reduced by 0.8% $K_4Fe(CN)_6$ (Sigma Aldrich) in 0.15 M Na-cacodylate buffer. Samples were contrasted with 1% uranylacetate (EMS Science) in diH2O, dehydrated in a graded series of acetone and embedded in Spurr's resin (EMS Science). Longitudinal, ultrathin sections (65–80 nm) were cut from the resin-embedded blocks with a diamond knife (Diatome-US, USA) using a Leica UCT ultramicrotome and caught on a copper grid covered with formvar film. Images of longitudinal oriented fibers were obtained via an FEI Tecnai 12 TEM fitted with an AMT XR-111 10.5 Mpx CCD camera at 3,200 – 15,000× magnification (80 kV). Morphometric analysis of mitochondria was performed using ImageJ (NIH). To obtain general information about mitochondrial morphology and abundance (the area of mitochondria and perimeter), the masks of individual mitochondria, nucleus and cell area were drawn. The percent of the sarcoplasmic area (cell area – nucleus area) covered by mitochondria was used for determination of mitochondrial area. Masks of individual mitochondria were used to generate data about mitochondria perimeter and circularity.

Full length blots

Figure 2A

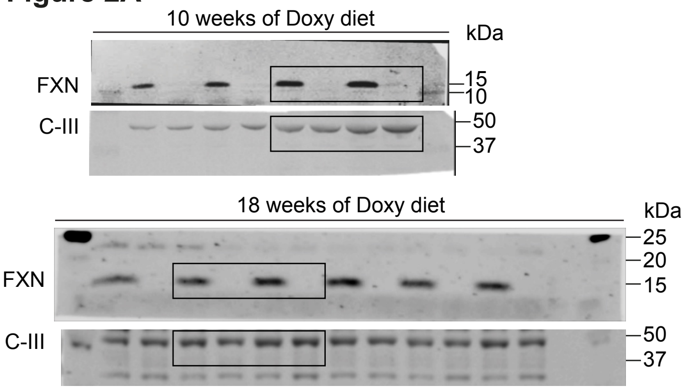

Figure 2B

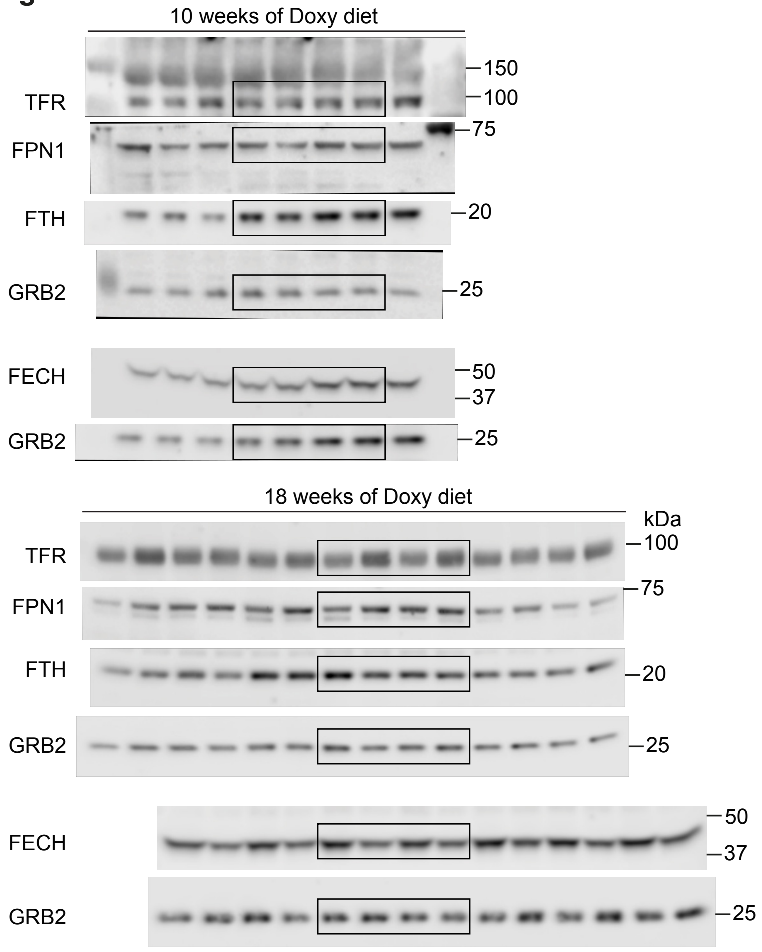

Figure 4A

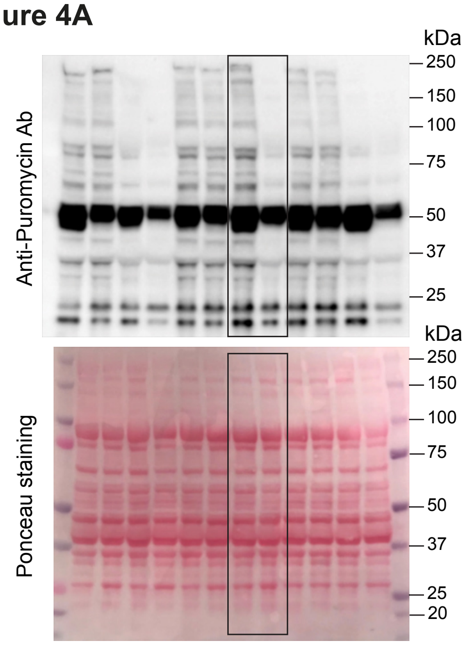

Figure 4C

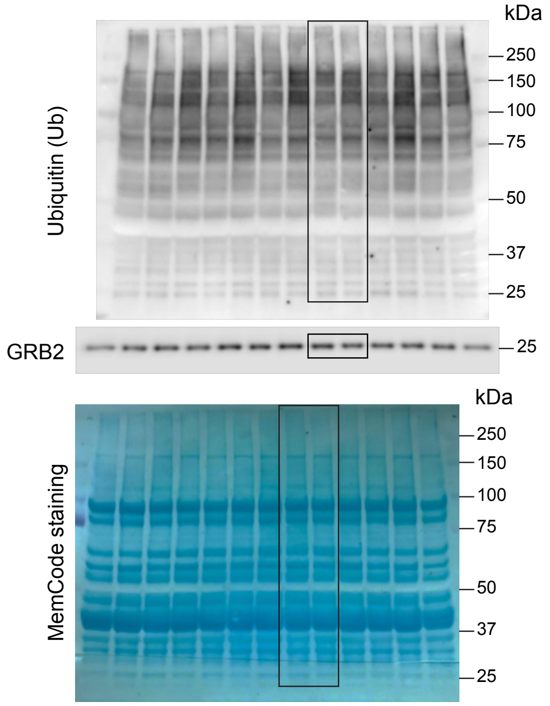

Figure 4C

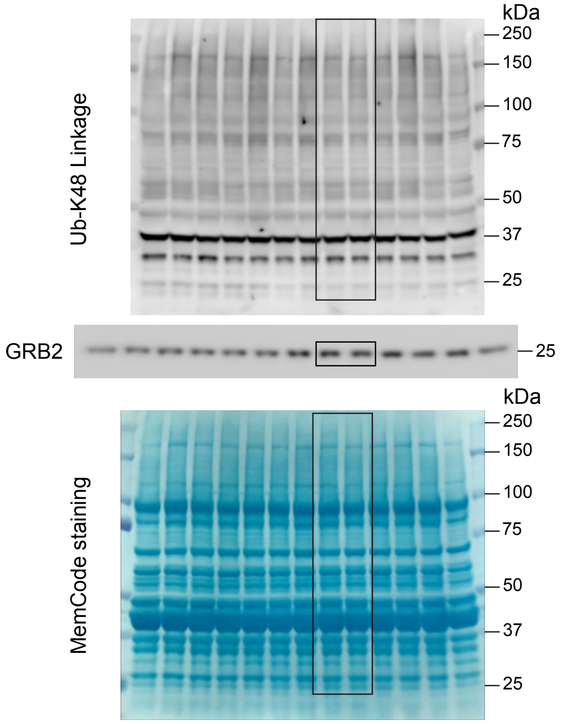

Figure 4D

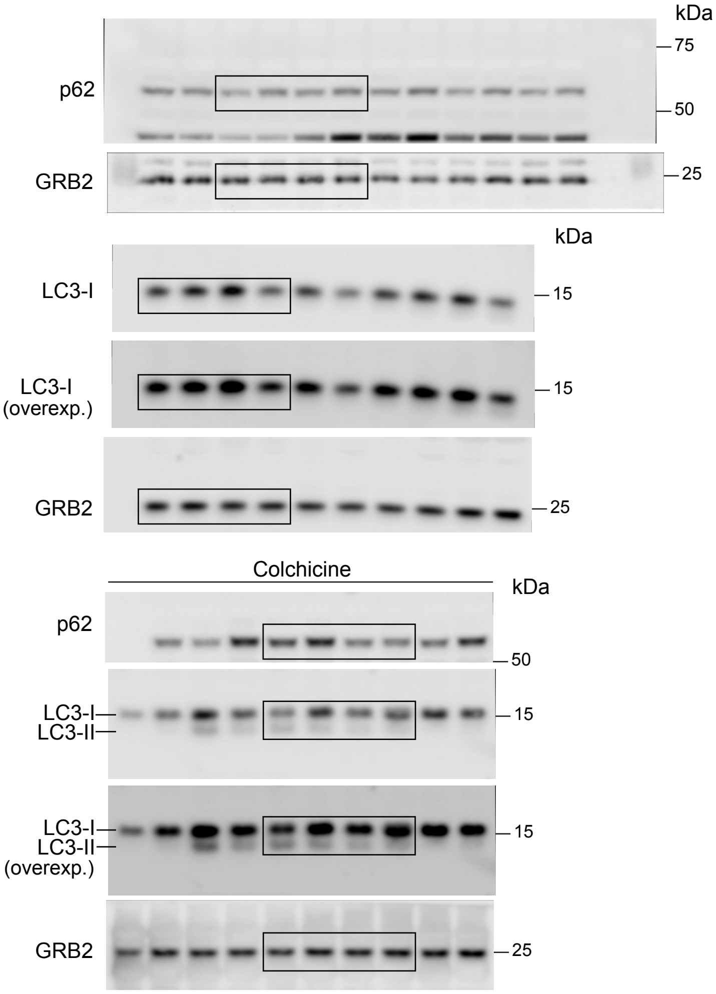

Figure 5A

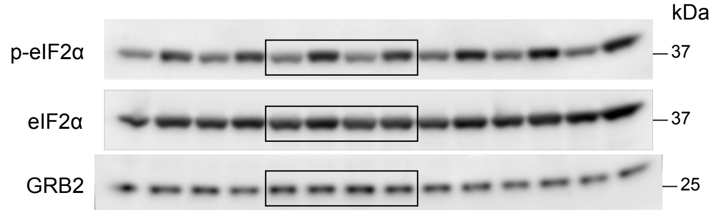

Figure 5B

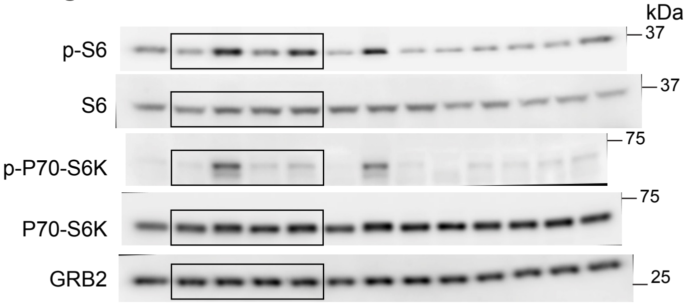

Figure 5C

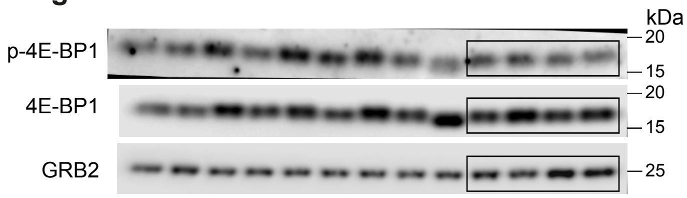

Figure 5D

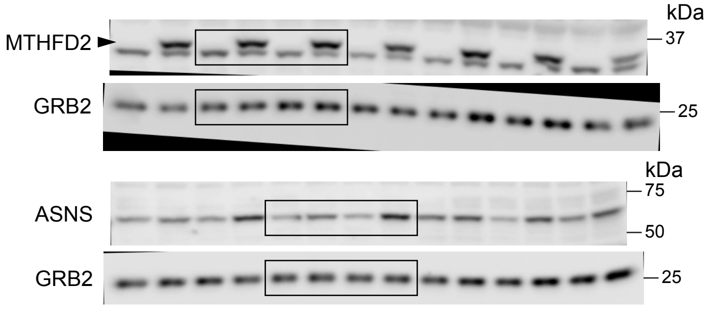

Figure 6C

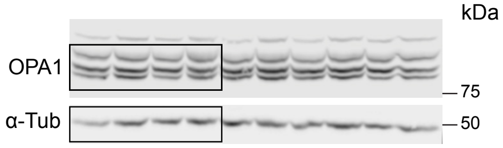

Figure 7F

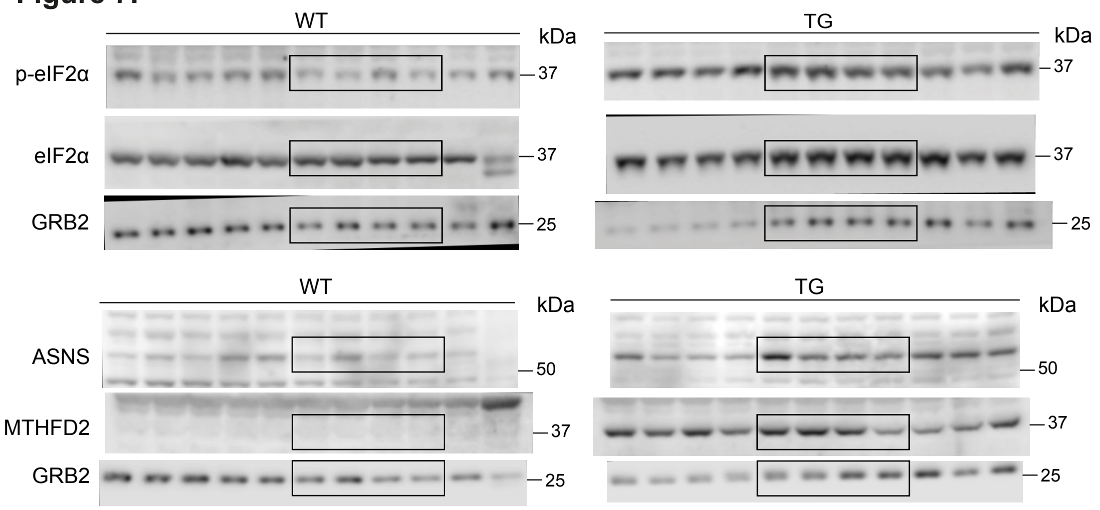

Figure 7H

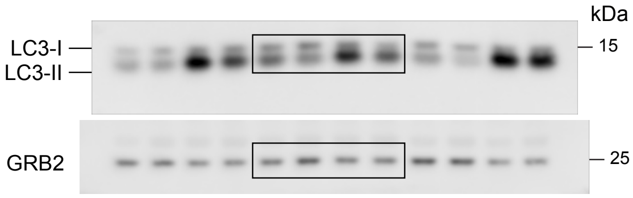

Suppl. Figure 3B

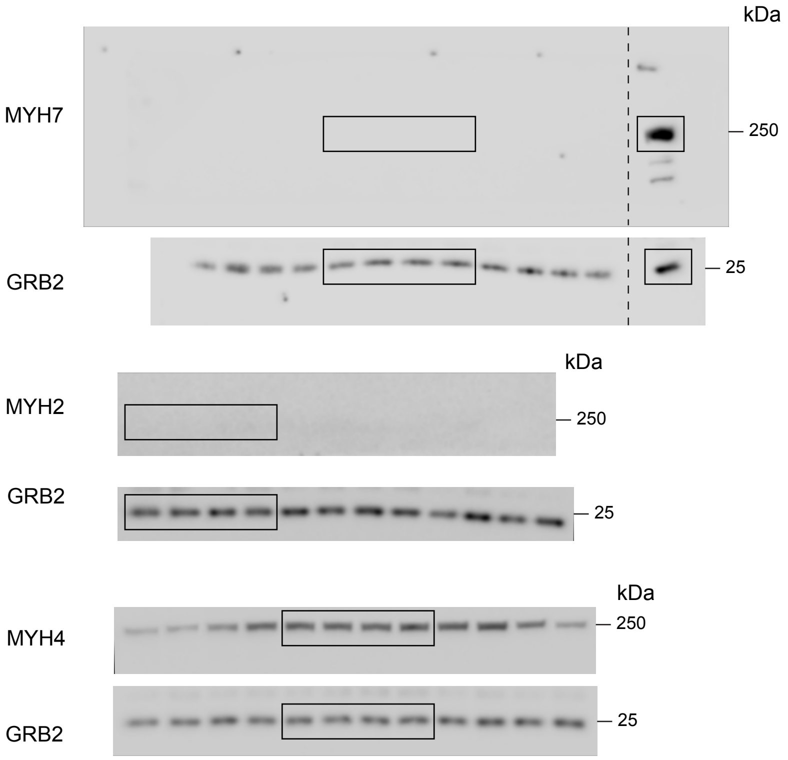

Suppl. Figure 4B

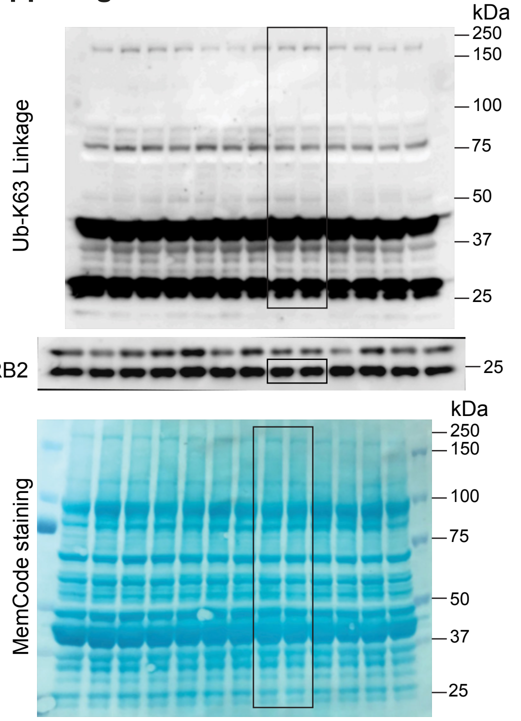

Suppl. Figure 5

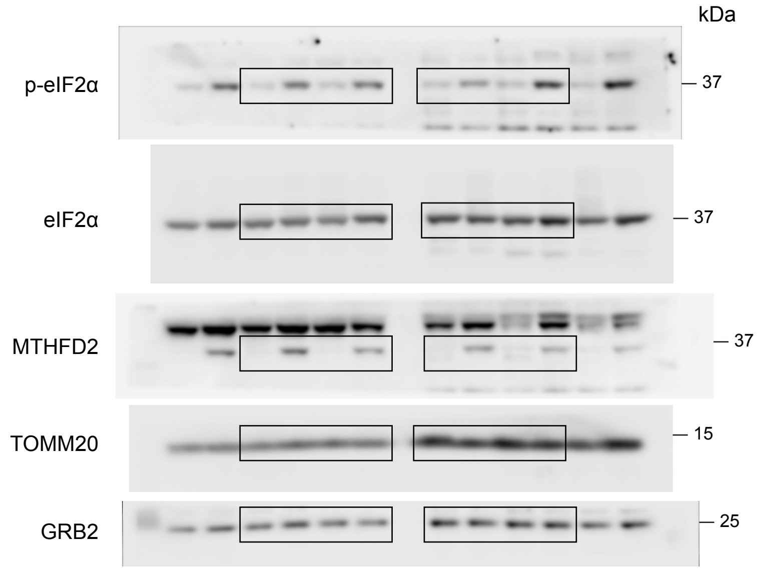

Suppl. Figure 6A

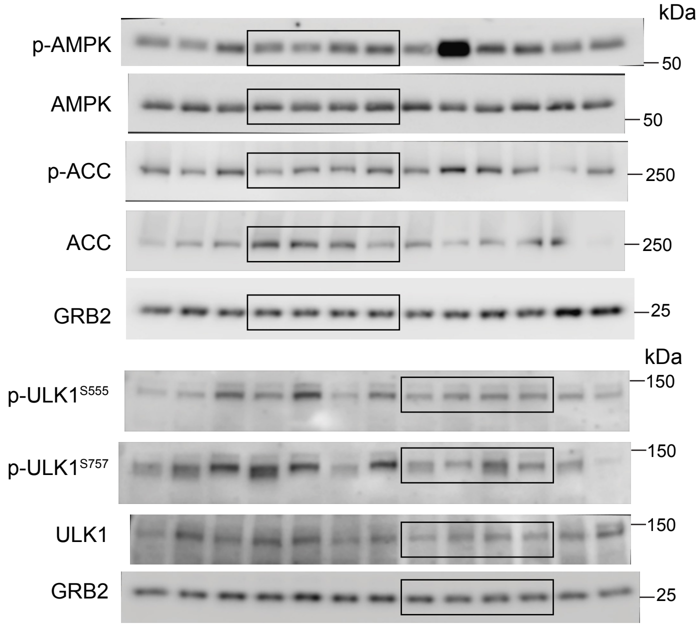

Suppl. Figure 6B

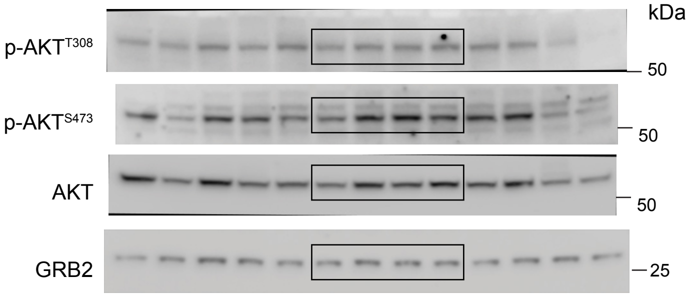

Suppl. Figure 6C

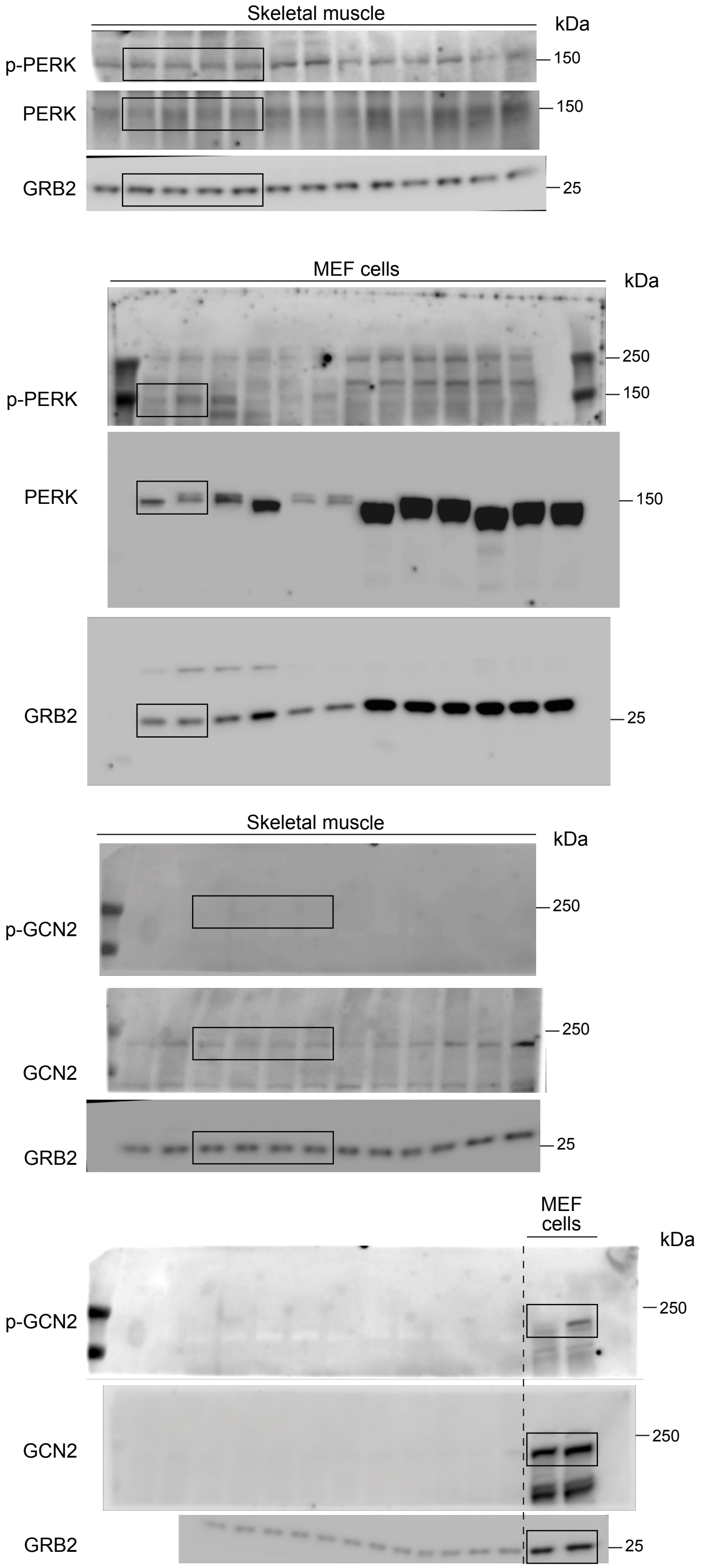

Suppl. Figure 6C

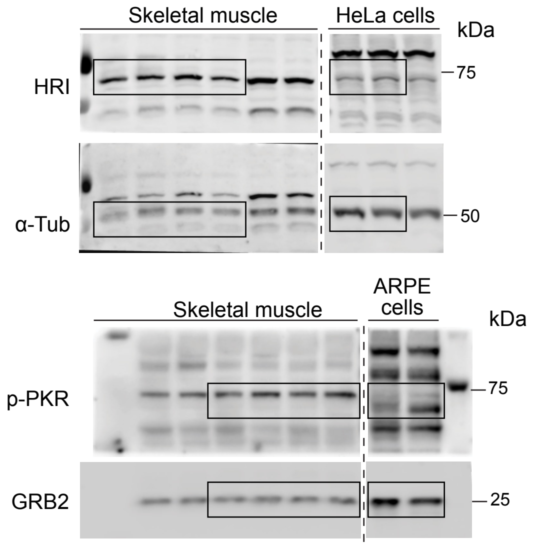

Suppl. Figure 6D

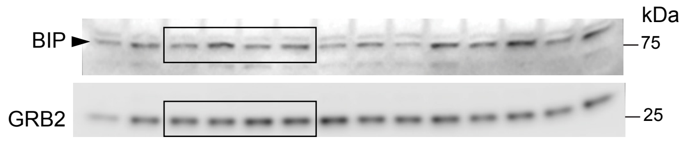

Suppl. Figure 7B

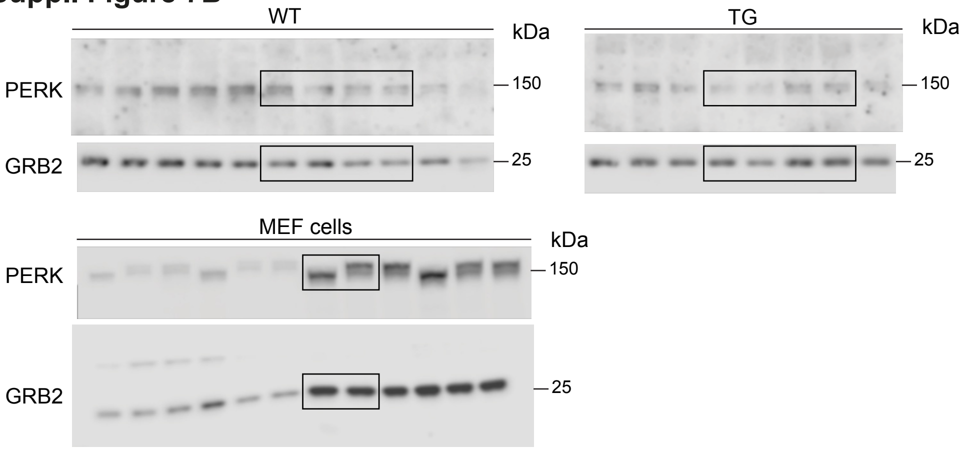

Suppl. Figure 7C

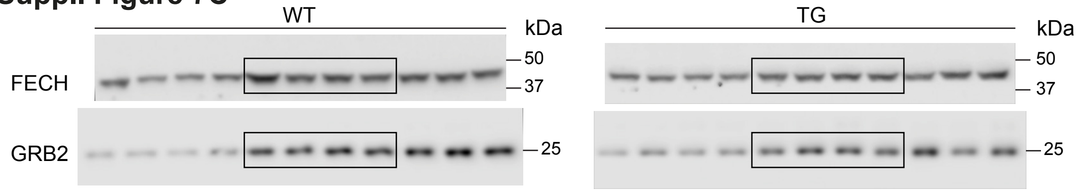

Suppl. Figure 7D

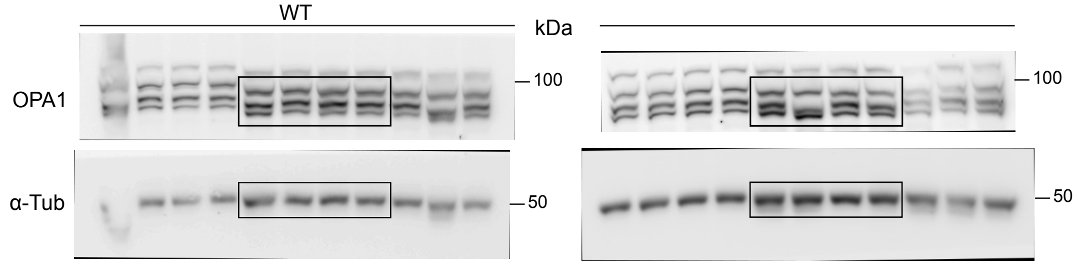

Supplement: Supplemental data [file jciinsight-7-155201-s236.pdf]
